# Proteomic screening identifies the zonula occludens protein ZO-1 as a new partner for ADAM12 in invadopodia-like structures

**DOI:** 10.18632/oncotarget.25106

**Published:** 2018-04-20

**Authors:** Bassil Dekky, Michael Ruff, Dominique Bonnier, Vincent Legagneux, Nathalie Théret

**Affiliations:** ^1^ Univ Rennes, Inserm, EHESP, Irset (Institut de recherche en santé, environnement et travail)-UMR_S1085, Rennes, France; ^2^ Present address: Institute of Biology Valrose (iBV) CNRS UMR7277 - INSERM U1091 – UNS University Nice Sophia Antipolis, Nice, France

**Keywords:** ADAM12, ZO-1, invadopodia, EMT, cancer

## Abstract

The epithelial mesenchymal transition (EMT) is a key process for cancer cell invasion and migration. This complex program whereby epithelial tumor cells loose polarity and acquire mesenchymal phenotype is driven by the regulation of cell-cell adhesion and cell-substrate interactions. We recently described the association of ADAM12 with EMT and we now use immunoprecipitation and proteomic approaches to identify interacting partners for ADAM12 during EMT. We identify twenty proteins that are involved in molecular mechanisms associated with adhesion/invasion processes. Integrative network analyses point out the zonula occludens protein ZO-1, as a new potential partner for ADAM12. *In silico* screening demonstrates that ZO-1 and ADAM12 are coexpressed in breast cancer cell lines sharing EMT signature. We validate the interaction between ZO-1 and ADAM12 in invasive breast cancer cell lines and show that ZO-1 and ADAM12 co-localize in actin- and cortactin-rich structures. Silencing either ADAM12 or ZO-1 inhibits gelatin degradation demonstrating that both proteins are required for matrix degradation. We further show that matrix metalloprotease 14, known to mediate degradation of collagen in invadopodia-like structures interacts with ZO-1. Depletion of PKCε that regulates the recruitment of ADAM12 and ZO-1 to cell membranes induces a decrease in ADAM12 and ZO-1 at invadopodia-like structures and degradation activity. Together our data provide evidence for a new interaction between ADAM12, a mesenchymal marker induced during TGF-β-dependent EMT and ZO-1, a scaffolding protein expressed in tight junctions of epithelial cells, both proteins being redistributed at the invadopodia-like structures of mesenchymal invasive cells to promote PKCε-dependent matrix degradation.

## INTRODUCTION

The epithelial-mesenchymal transition (EMT) plays a pivotal role during tumor progression and invasion allowing epithelial cells to acquire a migratory phenotype. This complex program occurs through numerous transitional states, some of them being reversible and characterized by cell heterogeneity [1]. Major hallmarks of EMT include the loss of tight junctions and the increase of mesenchymal markers expression such as vimentin and N-cadherin [2]. While overexpression of members of the disintegrin and metalloprotease (ADAM) family has been widely documented in cancers and invasion [3], few have been directly involved in EMT. SiRNA-based knockdown of ADAM9 in pancreatic cancer cells has been shown to diminish cellular migration, invasion, and to induce the epithelial marker E-cadherin [4] and a role of ADAM17 in promoting EMT has been recently reported in lung adenocarcinoma cells [5] and gastric carcinoma cells [6]. The association of ADAM12 with breast cancer aggressiveness and EMT has been suggested by several lines of evidence that include the ability of ADAM12-overexpressing breast cell lines to induce metastasis *in vivo* [7, 8] and its correlated expression with the presence of metastases in triple-negative breast cancer [9] and with a breast tumor-initiating cell phenotype [10].

ADAM12 exists as two spliced isoforms that give rise to a membrane-anchored long form ADAM12L and a shorter secreted ADAM12S form. We recently demonstrated that overexpression of ADAM12L, but not ADAM12S is sufficient to induce loss of cell-cell contact, reorganization of actin cytoskeleton, up-regulation of EMT markers and chemoresistance [11]. Although the proteolytic activity of the short isoform ADAM12S is required for cell migration and invasion [8], ADAM12L induces EMT through a protease-independent manner but requires the cytoplasmic tail [11]. Fifteen proteins have been previously reported to physically interact with ADAM12L including cell surface proteins such as integrin [12, 13], syndecan [14] and the type II transforming growth factor-β receptor TGFBR2 [15]. Other proteins include signaling proteins such as Src-family non-receptor tyrosine kinases SRC and YES [16], the adapter proteins GRB2 [16] and SH3PXD2A (FISH) [17], the regulatory subunit of phosphatidylinositol 3-kinase, PIK3R1 (p85α) [18], the protein kinases PRKCE [19] and PRKCD [18], and their receptor, RACK1 [20] and the integrin-linked kinase, ILK [21]. The interaction of ADAM12L with actin cytoskeleton and vesicle formation was further documented by the identification of two actin-related proteins, ACTN1 and 2 (α-actinin-1 and −2) [22] and the cytoplasmic PACSIN3 phosphoprotein [23]. Most of these proteins are common to all cells and have been already implicated in cell signaling associated with EMT.

In the present study, we searched for new interacting partners of the membrane-anchored ADAM12L long form in a specific ADAM12L-induced EMT model. Using mass-spectrometry (MS)-based proteomic approaches and integrative data mining of ADAM12L protein networks, we identified the zonula occludens protein ZO-1 encoded by TJP1 gene, as a new potential partner for ADAM12L. We validated this interaction and further demonstrated that endogenous ZO-1 and ADAM12L were co-localized in invadopodia-like structures and were required for matrix degradation in invasive cell lines, which exhibit a full mesenchymal phenotype. Importantly silencing PKCε impaired ZO-1 and ADAM12L distribution and totally abolished matrix degradation in invadopodia-like structures thereby providing evidence for a new functional interaction between ADAM12, ZO-1 and PKCε.

## RESULTS

### Identification of ZO-1 as part of ADAM12L protein interaction network

We recently demonstrated that forced expression of ADAM12L but not ADAM12S in the non-tumorigenic epithelial cell line MCF10A induced EMT [11]. In order to identify new functional partners of ADAM12 during this process, the anti-ADAM12L immunoprecipitates from ADAM12L-overexpressing MCF10A cells were size-separated by SDS-PAGE and in-gel digests were analyzed by LC-MS/MS, followed by protein identification through database searching. 253 and 200 proteins were identified in ADAM12L and IgG immunoprecipitates, respectively. When comparing the two conditions, 67 proteins were only detected in ADAM12L-immunoprecipitates (Supplementary Table 1). In order to discard contaminating proteins identified after immunoprecipitation, we submitted the list of proteins to the Contaminant Repository for Affinity Purification (CRAPome) [24] and sorted by the fold change scores to identify high-scoring interactions. The 20 retained proteins (shown in Table 1), are mostly implicated in molecular mechanisms associated with adhesion/invasion processes such as cytoskeleton remodeling and membrane trafficking (SYNE2 [25], AP2A1 [26], MIA2 [27]), PI3K-AKT signaling pathway (PLEKHA5 [28], ITPR3 [29]), cell junctions (SPECC1L, ZO-1, ZO-2) [30, 31] and regulators of these processes (NFAT5 [32], TBC1D15 [33]). Similarly Dock10 has been shown to affect adhesion and induce protrusion through Rac1 and Cdc42 activation [34, 35]. In addition, USP25 is a direct target of miR200C a known regulator of EMT [36] and PDE6H has been previously implicated with ADAM12 in cytotrophoblasts invasion [37].

**Table 1:** List of proteins specifically identified in ADAM12 immunoprecipitates and filtered using CRAPOME tool

GENE SYMBOL	ENTREZ-GENE	GENE NAME
ADAM12	8038	ADAM metallopeptidase domain 12
USP25	29761	Isoform USP25m of Ubiquitin carboxyl-terminal hydrolase 25
PDE6H	5149	phosphodiesterase 6H
DOCK10	55619	dedicator of cytokinesis 10
NFAT5	10725	nuclear factor of activated T-cells 5, tonicity-responsive
CIT	11113	citron rho-interacting serine/threonine kinase
SYNE2	23224	spectrin repeat containing, nuclear envelope 2
RNPEP	6051	Aminopeptidase B
PLEKHA5	54477	pleckstrin homology domain containing A5
ITPR3	3710	inositol 1,4,5-trisphosphate receptor type 3
MIA3	375056	melanoma inhibitory activity family member 3
SPECC1L	23384	CYTSA protein
C5	727	complement component 5
AFG3L2	10939	AFG3 like matrix AAA peptidase subunit 2
TBC1D15	64786	TBC1 domain family member 15
AP2A1	160	Isoform B of AP-2 complex subunit alpha-1
PRDX5	25824	peroxiredoxin 5
TJP2	9414	tight junction protein 2
TJP1	7082	tight junction protein 1
NUP205	23165	nucleoporin 205kDa

**Figure 1:**
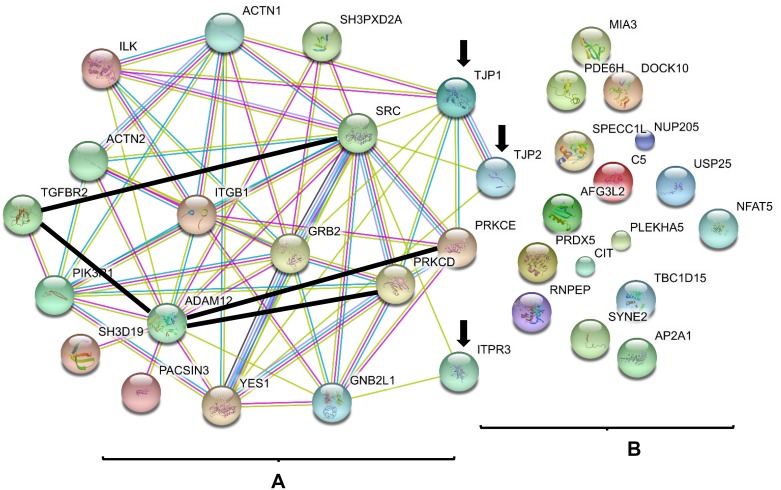
ADAM12 interactome The list of known proteins interacting with ADAM12L (**A**) and the list of new proteins identified in the present study (**B**) were used as input for STRING application. The graph was generated using default parameters. The annotation provided by STRING tool describes the different types of protein interactions. For known interactions: pink, experimentally determined; turquoise, from curated databases. For others interactions: olive green, textmining; black, co-expression; purple, protein homology. The interactions between ADAM12L and PRKCE [45], PRKCD [18], TGFBR2 [15] and between TGFBR2 and Src [71] have not been entered in the database yet and were added manually (thick black lines). Arrows indicated ADAM12L-interacting proteins identified by proteomic screen (B) that were connected with previous published ADAM12L interactome (A). TJP1 and TJP2 genes encode ZO-1 and ZO-2 proteins.

Because 15 other proteins have been previously identified as ADAM12L-binding proteins (listed in Supplementary Table 2), we thought to search for their functional interaction with the 20 proteins newly identified in ADAM12L-overexpressing MCF10A cells. For that purpose, the 15 validated proteins and the new 20 potential interacting proteins were analyzed together using STRING (Search Tool for the Retrieval of Interacting Genes/Proteins) tool [38] to generate protein-protein interaction networks. As shown in Figure 1, the 15 known ADAM12L interacting proteins are highly connected as previously described [21] but only three of the newly identified proteins showed interactions with this network: inositol 1,4,5-trisphosphate receptor type 3 (IPTR3) and the two tight junction-associated protein (ZO-1 and ZO-2). Of note, IPTR3 belongs to the Phosphatidylinositol signaling system as well as PI3KR1, a well-known partner of ADAM12 [39]. More interestingly, ZO-1 displayed interactions with six members of ADAM12L networks that include PRKCD, PRKCE, ILK, YES, SRC and GRB2 and has been previously implicated in EMT [40]. While association scores are mainly due to Co-citation in literature, experimental/biochemical data further support the interactions between ZO-1 and SRC or ILK (Supplementary Table 3). We therefore decided to study the functional interaction between ADAM12 and ZO-1.

### ADAM12L interacts with ZO-1 in ADAM12L-overexpressing MCF10A cells

**Figure 2:**
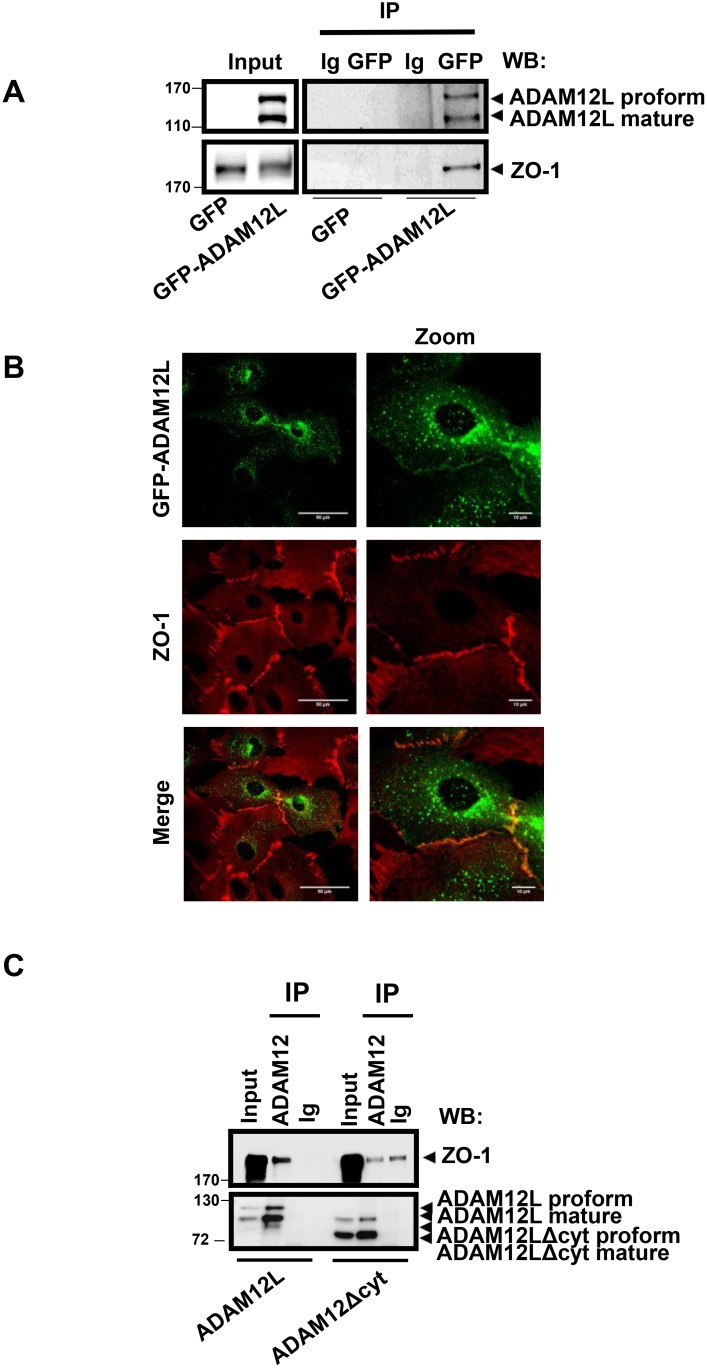
ADAM12L interacts with ZO-1 in ADAM12-overexpessing MCF10A cells MCF10A cells were infected with lentiviruses expressing either GFP-ADAM12L fusion protein (ADAM12L) or the control protein GFP (Control) as previously described [11]. (**A**) Crude extracts from GFP- or GFP-ADAM12L-overexpressing MCF10A were immunoprecipitated (IP) with anti-GFP antibodies or control IgG and immunoblotted with anti-ADAM12 or anti-ZO-1 antibodies (WB). (**B**) GFP-ADAM12L was detected as green staining and ZO-1 was immunolocalized and revealed in red. (**C**) Cos-7 cells were transfected with either ADAM12L, or a truncated form lacking the cytoplasmic domain (ADAM12-Δcyt). Crude extracts were immunoprecipitated (IP) with anti-ADAM12 antibodies or control IgG and immunoblotted with indicated antibodies (WB).

We first validated this interaction by immunoprecipitation studies in the GFP-ADAM12L-overexpressing MCF10A cells used for the affinity proteomic study. As shown in Figure 2A, endogenous ZO-1 was immunoprecipitated from extracts of these cells using GFP antibodies while no signal was recovered in GFP-overexpressing MCF10A used as control. Both pro- and processed GFP-ADAM12L, corresponding to the 110 and 90 kDa forms of endogenous protein, were detected. Of note, the reverse immunoprecipitation using anti-ZO-1 antibodies did not permit to immunoprecipitate ADAM12 in our conditions (data not shown). We further confirmed this interaction by investigating whether ADAM12 co-localized with ZO-1 in GFP-ADAM12L-overexpressing MCF10A (Figure 2B). We observed some immunostaining for ADAM12 and ZO-1 at the junctional interface between cells. Such colocalization pictures were rare because ADAM12L overexpression in MCF10A cells leads to mesenchymal phenotype characterized by decrease in cell-cell junctions [11] and because overexpressed ADAM12 in cells is mainly retained intracellularly [41]. To characterize the domain of ADAM12 that is required for interaction with ZO-1, Cos7 cells were transfected with either ADAM12L, or a truncated form lacking the cytoplasmic domain (ADAM12-Δcyt). As shown in Figure 2C, immunoprecipitation of ADAM12L led to the recovery of substantial amounts of ZO-1 while immunoprecipitation of ADAM12L-Δcyt led to amount similar to that of control immunoglobulins, pointing out the implication of the ADAM12 cytoplasmic domain in the interaction with ZO-1.

Together these data support evidence for interaction of ADAM12L with ZO-1 and question about the relationships between ADAM12, a known marker of mesenchymal cell differentiation and ZO-1, a marker of tight junctions in epithelia cells.

### ADAM12L and ZO-1 are co-expressed in invasive breast cancer cells

**Figure 3:**
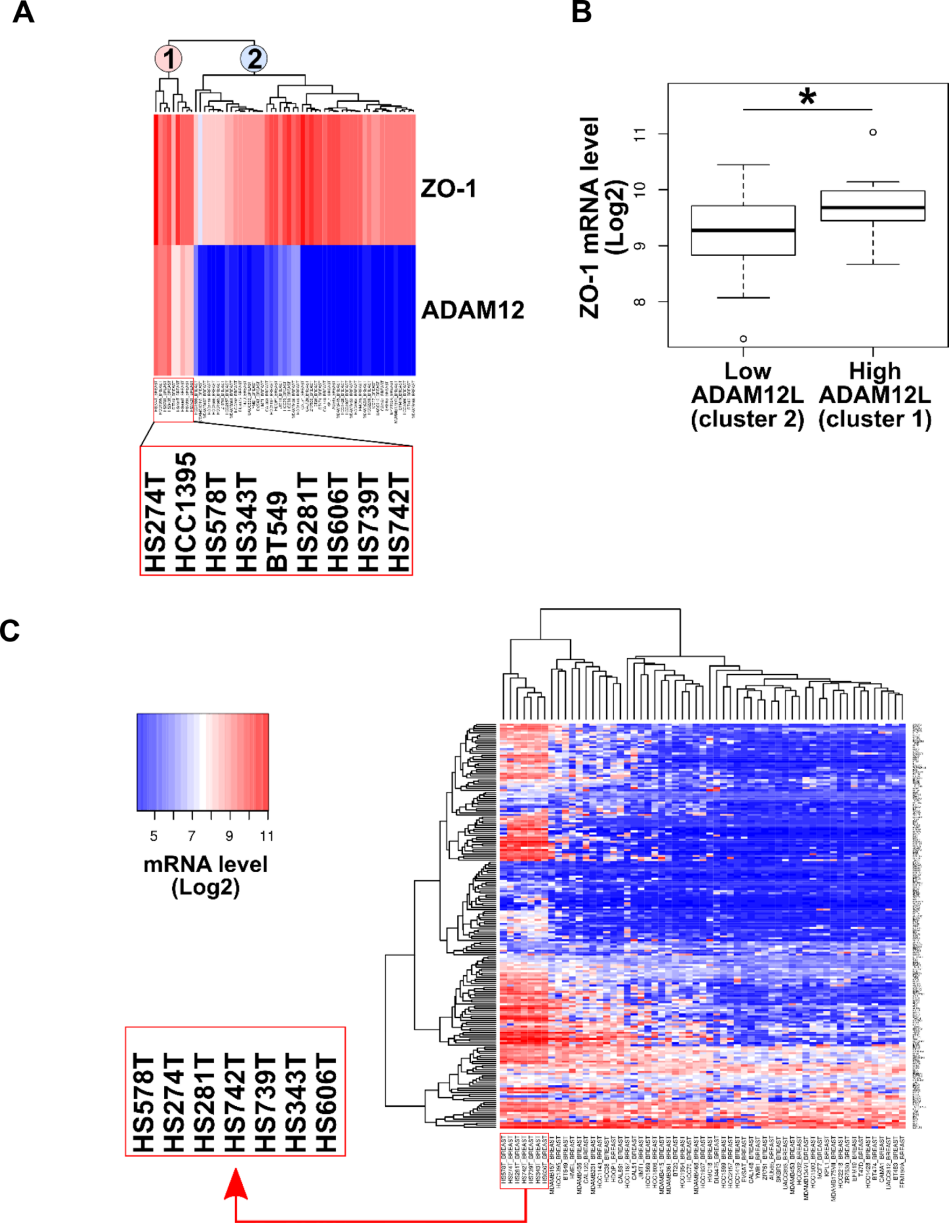
ADAM12L and ZO-1 gene expression in a panel of 59 breast cancer cell lines Breast cancer data (59 cell lines) were extracted from Cancer Cell Line Encyclopedia (Broad Institute). Data are expressed as gene-centric RMA-normalized mRNA expression data. (**A**) Hierarchical clustering analysis of breast cancer cell lines based on ADAM12L and ZO-1 expression allows to define two clusters, corresponding to high (cluster 1) and low (cluster 2) ADAM12 expression. Clusters 1 and 2 are indicated by pink and light-blue labels, respectively. (**B**) Comparative analysis of ZO-1 mRNA levels between breast cancer cell lines expressing high (cluster 1) and low (cluster 2) ADAM12L mRNA levels. (**C**) Hierarchical clustering analysis of breast cancer cell lines based on EMT gene signature. This clustering highlights cell lines with highest expression of EMT signature genes (boxed cell line names). Expression levels are coded by heatmap colors dark blue (low mRNA levels) to red (high mRNA levels), as indicated by the color key.

To demonstrate that endogenous ADAM12L interacts with ZO-1, we first searched for cell lines expressing both ADAM12 and ZO-1. For that purpose, we extracted gene expression data from 59 breast cancer cell lines selected from the Broad Institute Cancer Cell Line Encyclopedia [42, 43]. Unlike ADAM12L, ZO-1 is widely expressed by all breast cancer cell lines however, clustering analysis showed that ADAM12L and ZO-1 were highly co-expressed in 9 cell lines (HS274T, HCC1395, HS578T, HS343T, BT549, HS281T, HS606T, HS739T, HS742T) (Figure 3A). In addition, we showed that ZO1 expression is significantly increased in breast cancer cell lines characterized by high ADAM12L mRNA levels (*p <* 0.05) (Figure 3B). Note that ZO-2, a tight junction associated protein that interacts with ZO-1 did not show similar association with ADAM12L expression. Unlike ZO-1, cell lines with high-ADAM12 expression were enriched in low-ZO-2 (Supplementary Figure 1). Because we previously reported the association of ADAM12 expression with EMT signature, we performed a new clustering analysis of breast cancer cell lines based on the expression of EMT gene set from the Molecular Signatures Database (Supplementary Table 4). As shown in Figure 3C, this allowed to highlight a cluster of 7 cell lines with high expression levels of EMT gene markers (HS578T, HS274T, HS281T, HS343T, HS606T, HS739T, HS742T). Strikingly, all these cell lines exhibit high expression levels of both ADAM12L and ZO-1 (Figure 3A). Together these data showed that breast cancer cell lines with EMT phenotype expressed high levels of ADAM12L and ZO-1. Since a clear relationship between EMT gene expression and invasive potential of breast carcinoma cells has been well established [43], our observations indicate that co-expression of ZO-1 and ADAM12 is linked to invasiveness.

### ADAM12L interacts with ZO-1 in invadopodia-like structures

**Figure 4:**
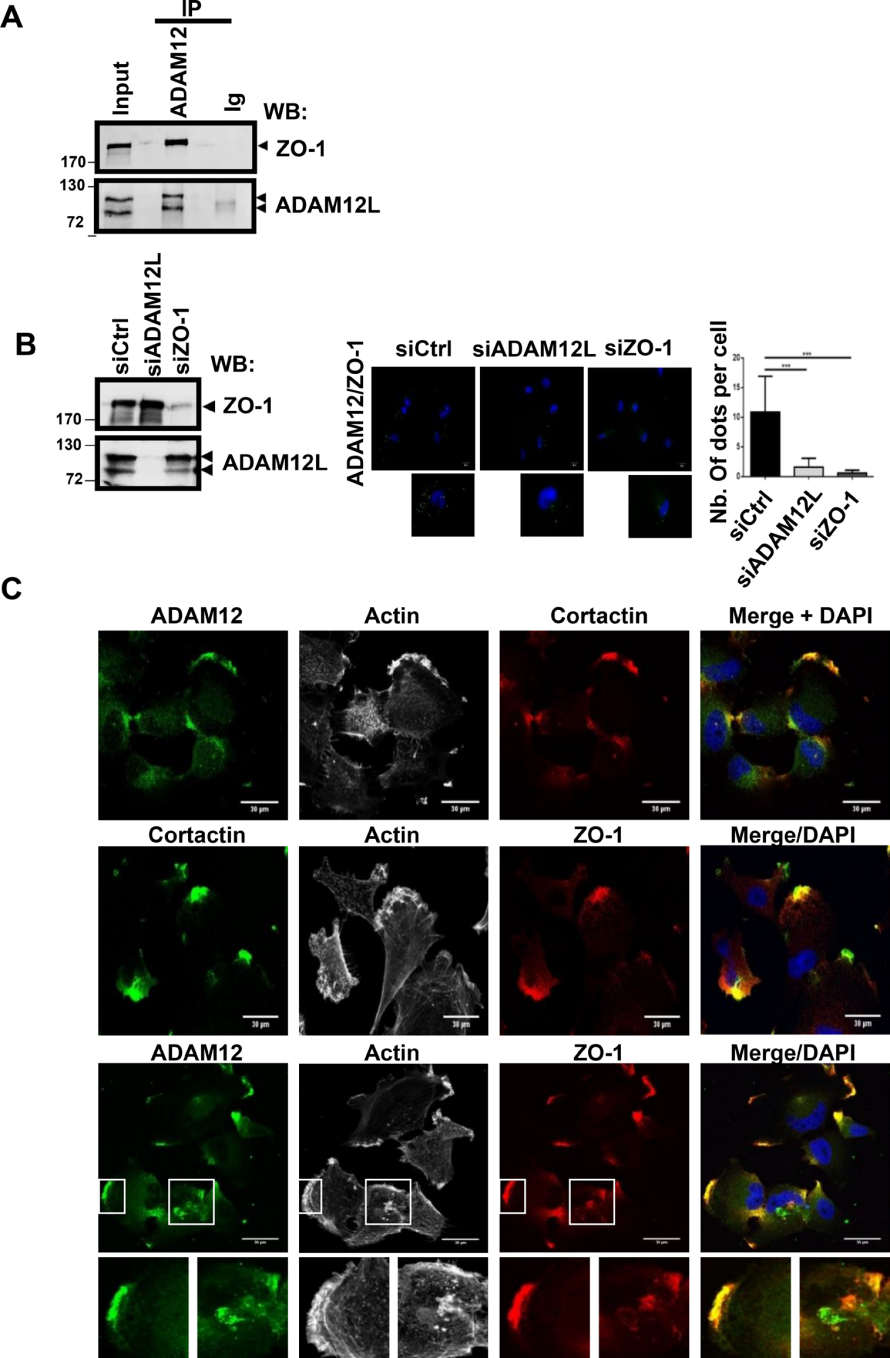
ADAM12L interacts and co-localizes with ZO-1 in breast cancer cell line HS578T (**A**) Crude extracts from HS578T cells were immunoprecipited with anti-ADAM12 antibodies or control IgG (IP) and immunoblotted with indicated antibodies (WB). (**B**) Interaction between ZO-1 and ADAM12 was analyzed by proximity ligation assay in HS578T cells silenced or not with either ADAM12L or ZO-1. Interaction results in fluorescent dots (green). Results are expressed as the mean ± SD of three independent experiments, (^***^P < 0.001). Representative fields are shown. Negative controls are samples with only primary or secondary antibodies (see Supplementary Figure 4). (**C**) HS578T cells were immunostained with antibodies against ADAM12 (green), ZO-1 (red) and Cortactin (red or green). Representative fields are shown. Co-localization of ADAM12 and ZO-1 results in yellow cellular staining. The actin cytoskeleton was stained by fluorescent phalloidins 547H (gray) and nuclei were stained with Hoechst 33258 dye (blue).

In accordance with *in silico* screening, we selected the invasive HS578T cell line that expressed the two proteins to validate the interaction between endogenously expressed ADAM12L and ZO-1. When immunoprecipitation was performed using anti-ADAM12 antibodies, ADAM12/ZO-1 complexes were clearly detected in cell extracts (Figure 4A). We observed similar interaction using another breast cancer cell line, BT549 (Supplementary Figure 2). The reverse immunoprecipitation using four different anti-ZO-1 antibodies did not permit to identify ADAM12L in immunoprecipitates (Supplementary Figure 3). This observation might be due to the lack of efficacy of ZO-1 immunoprecipitation associated or not with competitive effects between binding domains recognized by diverse ZO-1-interacting proteins and ZO-1 antibodies (Supplementary Figure 3). To explore the specificity of interaction between ADAM12 and ZO-1, we performed proximity ligation assays in cells, silenced for either ADAM12 or ZO-1 (Figure 4B and Supplementary Figure 4). To this purpose, we performed knock-down experiments in HS578T cells using small interfering RNA (siRNA) against ADAM12L and ZO-1. After 72 h of treatment with siADAM12L, the steady-state levels of ADAM12 protein were strongly reduced in HS578Tcells while ZO-1 levels were reduced by up to 80% in siZO-1 treated cells, indicating robust RNA interference efficiency (Figure 4B and Supplementary Figure 5). Proximity ligation assays showed numerous stained dots in HS578T cells treated with control siRNA supporting evidence for ADAM12-ZO-1 interaction. At the opposite, silencing cells for either ADAM12L or ZO-1 prevents fluorescent signal demonstrating the specificity of the interaction. Using immunolocalization analyses, we further demonstrated that ZO-1 and ADAM12 are co-localized in cortactin- and actin-positive structures (Figure 4C). These observations are in accordance with previous data reporting partial colocalization of ADAM12L with actin and cortactin in clusters of invadopodia-like structures from ADAM12L-overexpressing cells [44] and the interaction of ZO-1 with cortactin in colorectal invasive cells [45].

### Interaction of ADAM12L with ZO-1 is associated with matrix degradation and invasion

**Figure 5:**
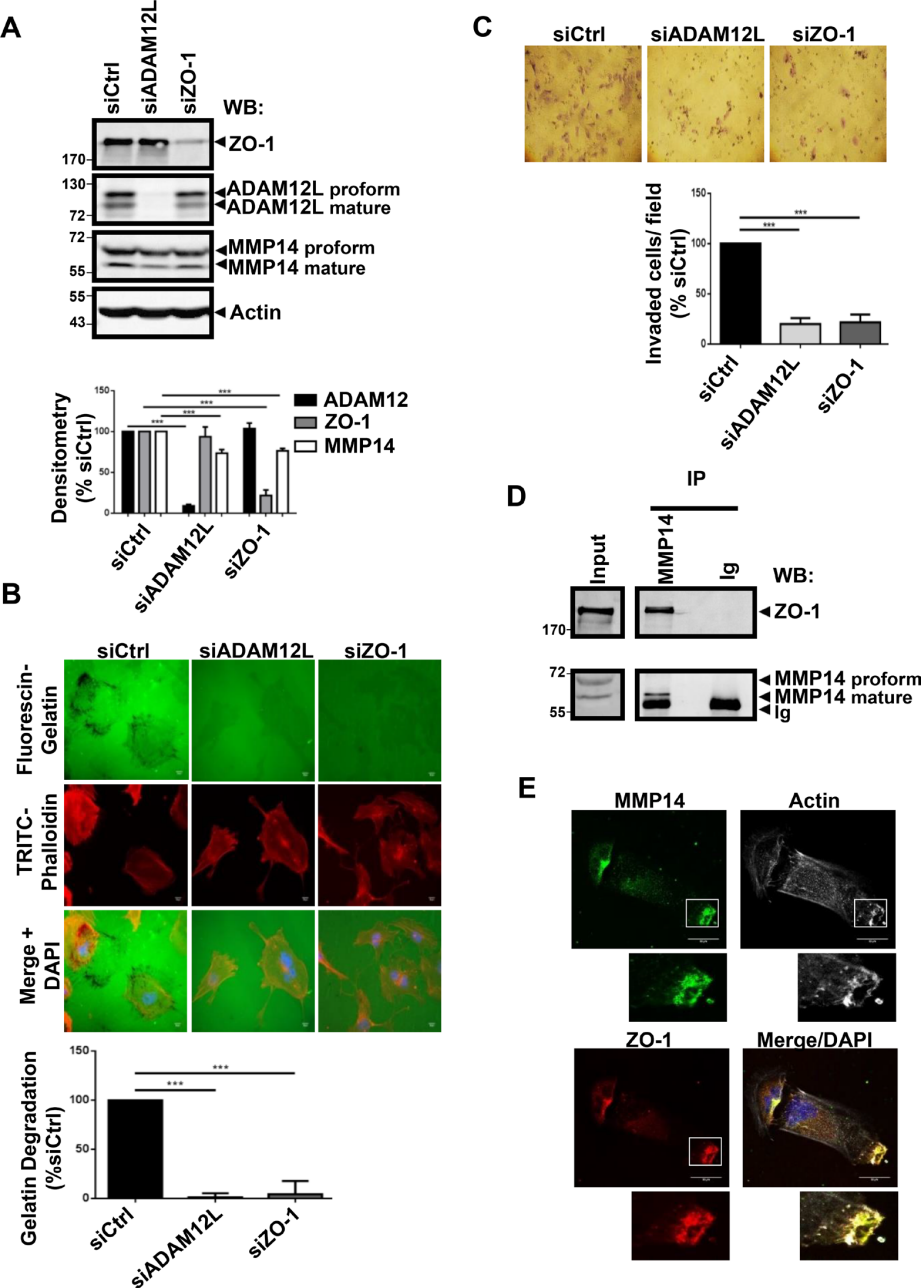
RNA interference–mediated decreased expression of ADAM12L and ZO-1 in HS578T cells abolished matrix degradation activity and invasion HS578T cells were transfected with 2 nM non-targeting siRNA (siCtrl) or ADAM12L siRNA (siADAM12) or ZO-1 siRNA (SiZO-1). (**A**) After 72 h, the efficiency and specificity of RNA interference were confirmed by western blotting. (**B**) Degradation activity was analyzed by using fluorescent gelatin. One representative picture is shown for each condition (Actin in red, Gelatin in green). Values of cells treated with control siRNA (siCtrl) were set as 100% of degradation. Results are expressed as the mean ± SD of three independent experiment, (^***^P < 0.001). (**C**) Matrigel invasion assay was performed using BioCoat™ Matrigel Invasion Chambers. Invading cells stained with Giemsa were counted in five different view fields, using NIH ImageJ software. Results are expressed as the mean ± SD of three independent experiments, (^***^P < 0.001). (**D**) Crude extracts from HS578T cells were immunoprecipitated with anti-MMP14 antibodies and immunoblotted with indicated antibodies. (**E**) Cells were immunostained with antibodies against MMP14 (green) and ZO-1(red). Representative fields are shown. Co-localization results in yellow cellular staining. The actin cytoskeleton was stained by fluorescent phalloidins 547H (gray) and nuclei were stained with Hoechst 33258 dye (blue).

Invadopodia and podosomes share many structural and functional features, among them the ability to degrade extracellular matrix through MMP14-dependent mechanisms [46]. We therefore asked whether modulating expression of endogenous ADAM12L and ZO-1 could influence MMP14 expression and gelatin degradation activity (Figure 5). While silencing ADAM12L and ZO-1 did not affect protein levels of each other, it induced a slight decrease in MMP14 protein levels (Figure 5A). In accordance with this observation, silencing ZO-1 has been previously reported to downregulate MMP14 expression in human breast cancer cells [47]. Importantly we showed that silencing ADAM12L and ZO-1 did not modify the localization of MMP14 (Supplementary Figure 6). Silenced cells for either ADAM12L or ZO-1 were plated on FITC-gelatin matrix for 16h and degraded areas were evaluated by image analyses. As shown in Figure 5B, degradation activity of HS578T cells was totally abolished by silencing either ADAM12L or ZO-1. The observation of ZO-1 requirement for degradation activity corroborates previous reports showing that matrix degradation is decreased upon ZO-1 silencing in HS578T cells [47] and macrophages [48]. Using gelatin-coated tissue culture transwell inserts we further showed that silencing either ADAM12L or ZO-1 prevented HS578T cells invasion (Figure 5C). To demonstrate interdependency between the individual effects of ADAM12L and ZO-1, we take advantage of the breast cancer cell line BT549 that is characterized by lower ADAM12 levels than HS578T. We demonstrated that overexpression of ADAM12L induced increase in gelatin degradation in a concentration-dependent manner that was inhibited by silencing ZO-1 (Supplementary Figure 7).

Together, these data are in agreement with the role of ADAM12L in activating MMP14 matrix protease, leading to gelatin degradation in ADAM12L-overexpressing cells [49]. We now showed for the first time that antibodies directed against MMP14 precipitated ZO-1 suggesting functional association between ZO-1 and MMP14 implicated in matrix degradation (Figure 5D). In support of this observation, we further co-localized ZO-1 and MMP14 in actin-rich structures (Figure 5E). Together our data support evidence for the involvement of complexes associating ADAM12L, ZO-1 and MMP14 in invadopodia-like structures of HS578T cells.

### Localization of ADAM12L and ZO-1 complexes in invadopodia-like structures and matrix degradation are dependent on PKCε

**Figure 6:**
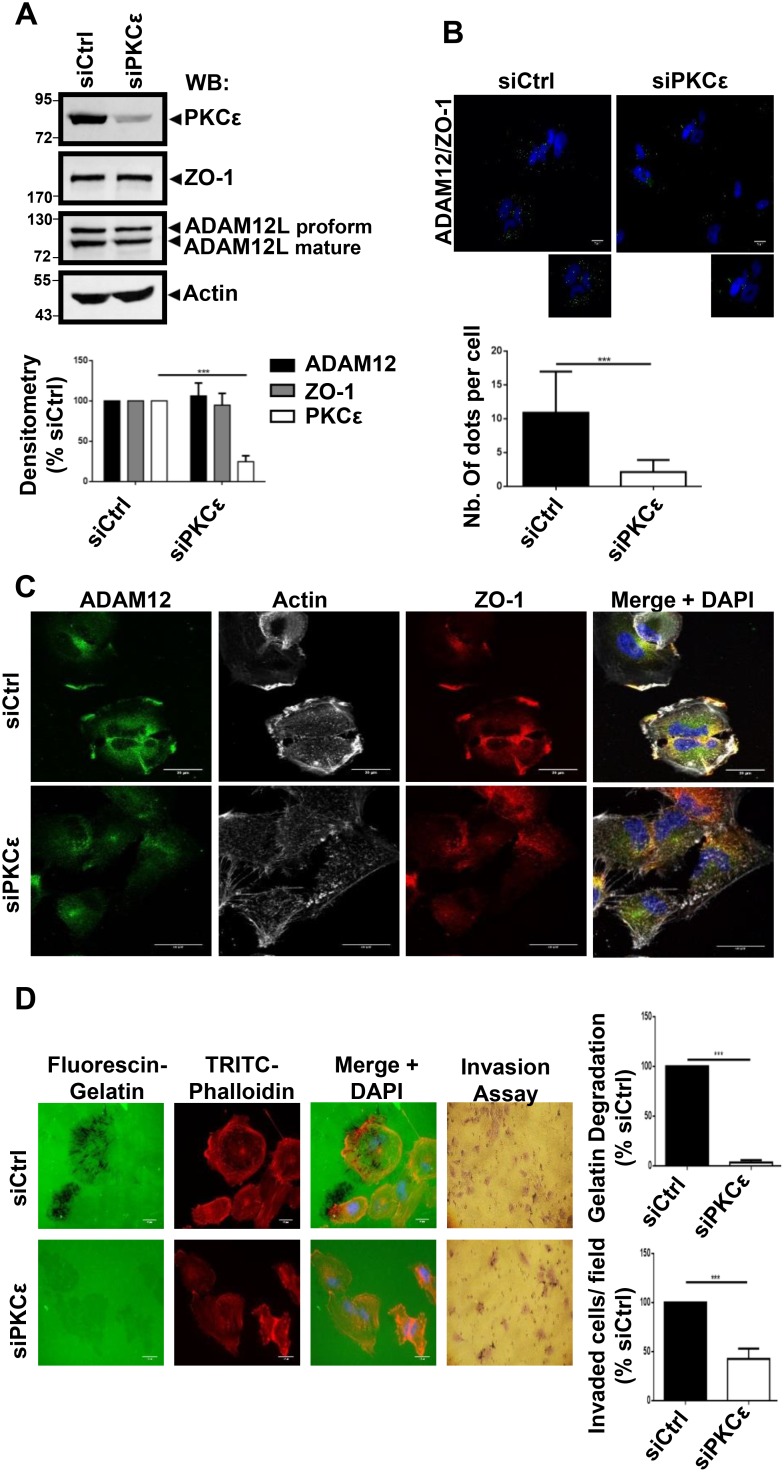
RNA interference–mediated decreased expression of PKCε in cells impaired ADAM12L and ZO-1 localization and totally abolished matrix degradation activity HS578T cells were transfected with 2 nM non-targeting siRNA (siCtrl) or PKCε siRNA (si PKCε). (**A**) After 72 h, Western blotting were used to confirm the efficiency and specificity of RNA interference in cell extracts. (**B**) Interaction of ZO-1 and ADAM12 was analyzed by proximity ligation assay in HS578T cells silenced for PKCε. Interaction results in fluorescent dots (green). Results are expressed as the mean ± SD of three independent experiments, (^***^P < 0.001). Representative fields are shown. Negative controls are samples with only primary or secondary antibodies (see Supplementary Figure 4). (**C**) Cells were immunostained with antibodies against ADAM12L and ZO-1 in PKCε silenced cells. Representative fields are shown. Co-localization results in yellow staining. The actin cytoskeleton was stained by fluorescent phalloidins 547H (gray) and nuclei were stained with Hoechst 33258 dye (blue). (**D**) Degradation activity was analyzed by using fluorescent gelatin. One representative picture is shown for each condition (Actin in red, gelatin in green). Values of cells treated with control siRNA (siCtrl) were set as 100% degradation. Results are expressed as the mean ± SD of three independent experiment (^***^P < 0.001).

During the course of immunofluorescence studies, we observed that silencing ADAM12L did not modify the amount of ZO-1 but the localization of ZO-1 was dramatically affected. We observed that ZO-1 staining was no more detected in the invadopodia-like structures while becoming diffusely cytoplasmic. At the opposite, silencing ZO-1 did not affect ADAM12L distribution (Supplementary Figure 8). These observations suggested that interaction between ADAM12L and ZO-1 plays a critical role in localization of ZO-1 to invadopodia-like structures. Interestingly, we and others have previously demonstrated the role of PKCε in regulating translocation of ADAM12L to the plasma membrane [19, 20] and recruitment of ZO-1 at the leading edge of lung cancer cells has been also shown to depend on PKCε [50]. In that context, we decided to explore the role of PKCε in regulating localization of ADAM12L and ZO-1 in invasive HS578T cells and its effect on matrix degradation. We performed PKCε knockdown in HS578T cells and after 72 h of treatment, the steady-state level of PKCε was significantly reduced while ADAM12L and ZO-1 expression was not modified (Figure 6A). Using proximity ligation assay, we further showed that interaction between ADAM12L and ZO-1 was dramatically diminished in PKCε-silenced cells compared to siCtrl transfected cells (Figure 6B). This observation was associated with the decrease of ZO-1 and ADAM12L staining in actin-rich structures, the localization of these proteins becoming diffusely cytoplasmic (Figure 6C). Together these results suggested that PKCε silencing modified ZO-1 and ADAM12L distribution supporting evidence for a pivotal role of PKCε in recruiting both ZO-1 and ADAM12 at invadopodia-like structures. Importantly we further showed that gelatin degradation activity and invasion were inhibited in HS578T cells silenced for PKCε (Figure 6D). Together these data were in agreement with a functional association between PKCε, ADAM12L and ZO-1.

## DISCUSSION

ADAM12 has been initially involved in differentiation of myoblast cells [51] and skeletal muscle regeneration [22]. Next, numerous studies converged to demonstrate the role of ADAM12 in differentiation of mesenchymal cells including chondrocytes [52], adipocytes [53], myoblasts [54] and osteoblasts [55]. More recently, the increased ADAM12 expression in cancer has been associated with EMT [10, 11]. All these observations suggest that ADAM12 plays a critical role in changes associated with cell differentiation.

To explore the molecular mechanisms involved in ADAM12L-mediated EMT, we performed a proteomic screening of proteins immunoprecipitated by anti-ADAM12 antibodies in ADAM12L-overexpressing epithelial breast cells. Functional annotation of the 20 proteins identified as potential new ADAM12L-interacting proteins revealed molecular mechanisms such as cytoskeletal remodeling, membrane trafficking, adhesion and invasion which are directly involved in EMT [56]. Of note, some of these proteins have been already related to known ADAM12L functions such as PI3K-AKT signaling [21] and cytotrophoblast invasion [37]. In order to explore how these proteins are connected to ADAM12L protein networks, we used an integrative data mining approach and identified the tight junction protein ZO-1 (encoded by TJP1) as being highly connected to the network of already known ADAM12L interacting proteins. Using immunoprecipitation and immunohistochemistry studies, we validated the interaction of ZO-1 with ADAM12 in different cell types. Although ZO-2 was also detected in the proteomic screen of ADAM12 immunoprecipitates from ADAM12L-overexpressing MCF10A cells, ZO-2 was never immunoprecipitated nor co-localized with endogenous ADAM12L in cells that constitutively expressed this protein (data not shown). Noteworthy, expression of ZO-2 is very low in cell lines expressing both ZO-1 and ADAM12L (Supplementary Figure 1). ZO-1 and ZO-2 proteins are well known to form complexes and are both critical to junction assembly in epithelial cells [57] and our proteomic screen was performed in the epithelial cell line MCF10A that are engaged into EMT upon ADAM12L over expression. One can hypothesize that complexes between ADAM12L and ZO-2 detected by proteomic screen in these cells came from an intermediate state where ZO-1 interacts with both ZO-2 (specific for epithelial state) and ADAM12L (specific for mesenchymal state). In line with these hypotheses, ZO-1 and ZO-2 independently contribute to embryonic development [58, 59] and formation of tight junctions [60] suggesting different roles.

During the course of EMT, dissociation of tight junctions is associated with the redistribution of ZO-1 from cell–cell contacts [61]. A few reports indicate that in cells having reached a fully mesenchymal phenotype, ZO-1 acquires new localizations and functions related to the invasive properties of these cells. ZO-1 has been found at the leading edges of lung cancer cells [50] and invading melanoma cells [62]. In accordance with this, the non-classical junctional functions of ZO-1 is further supported by its localization in non-epithelial cells including fibroblasts where it contributes to adhesion and migration [48, 62, 63].

Similarly, ADAM12 has been associated with invasion and we hypothesized that ADAM12L interacts with ZO-1 in invadopodia-like structures from cancer cells. In agreement with this hypothesis, extracellular engagement of ADAM12 has been shown to induce clusters of invadopodia [44] and the expression of ADAM12 has been associated with the formation of invadopodia in hypoxia [64]. We now demonstrated that ADAM12 co-localizes with ZO-1 and cortactin in actin-rich structures similar to invadopodia-like structures, and that localization of ZO-1 in these structures depends on ADAM12L. Our data further showed that silencing either ADAM12L or ZO-1 inhibited matrix degradation and invasion, hallmark features of invadopodia. In line with these observations, the expression of ADAM12 was recently associated with invadopodia formation and matrix degradation during Twist1-induced tumor invasion [65]. Importantly we demonstrated for the first time that ZO-1 interacts with MMP14, a major metalloproteinase implicated in matrix degradation. Because ADAM12 has been also shown to interact with MMP14 [49], we proposed that ADAM12L and ZO-1 act together for MMP14-mediated degradation in invadopodia-like structures. Of note, ADAM12 have been previously localized to podosomes of Src-transformed cells [17] and ZO-1 was recently proposed to modulate podosome formation [48]. Because podosome share many features with invadopodia, we verified that ADAM12 and ZO-1 co-localized to podosomes of Src-transformed NIH3T3 cells (Supplementary Figure 9).

Dynamics of EMT and formation of invadopodia require complex protein trafficking and we showed that silencing ADAM12L affects ZO-1 distribution. Similarly, PKCε silencing altered both ADAM12 and ZO-1 localization and abolished matrix degradation supporting evidence for functional ADAM12L-ZO-1-PKCε complexes during EMT. Such observations are consistent with the previous observations reporting PKCε-dependent distribution of ADAM12L towards membranes [19, 20] and ZO-1 to invadopodia [50]. Importantly, the present screening identified the Guanine nucleotide-exchange factor DOCK10 as a new interacting ADAM12L protein (Table 1). DOCK10 is responsible for activation of the small GTPase Cdc42 during cell invasion and the association of Cdc42 with cancer and EMT has been widely documented [66]. More recently Cdc42 requirement has been demonstrated for cell migration through the formation of ZO-1-MRCKβ (Cdc42BPB) complex at the leading edges of cells [67]. Together these observations suggest relationships between ADAM12-DOCK10 and ZO1-MRCKβ through Cdc42. Interestingly, PKCε activity has been involved in the regulation of Cdc42 [68] and might affect both ZO-1-MRCKβ and DOCK10-ADAM12 complexes that bind Cdc42. Together these data strengthened the functional association of ADAM12L, ZO-1 and PKCε the later regulating protein trafficking through RhoGTPase activities.

To conclude, we identified ZO-1 as a new binding partner for ADAM12L in invadopodia-like structures and we show that both proteins contribute to matrix degradation and invasion. This interaction is specific of ZO-1 since ZO-2 did not show similar function. Localization of both ADAM12L and ZO-1 is modulated by PKCε that participates to protein trafficking within the cell. Further investigations are required to decipher the molecular mechanisms by which ADAM12L, that is induced during the EMT, targets ZO-1 to the cellular structures involved in invasion.

## MATERIALS AND METHODS

### Proteomics analyses

Immunoprecipitation of proteins associated with ADAM12L were performed using extracts from ADAM12L-overexpressing MCF10A cells and immunoprecipitation using Rabbit IgG was included as control. Proteins from immunoprecipitates were denaturated and size-separated by sodium dodecyl sulfate-polyacrylamide gel electrophoresis (SDS-PAGE). After cutting gel tracks in 10 bands, in-gel digestion of proteins was performed with trypsin and resulting peptides were injected into a capillary HPLC system coupled to a mass spectrometer via a nanospray ionization source (ES MS/MS). All MS/MS samples were analyzed using Mascot (Matrix Science, London, UK; version 2.4.1) and X! Tandem softwares. Mascot was set up to search the TAX_HomoSapiens_9606_20141128 database. X! Tandem was set up to search a subset of the TAX_HomoSapiens_9606_20141128 database. For protein identification, Scaffold software (version 4.4.1.1, Proteome Software Inc., Portland, OR) was used to validate MS/MS based peptide and protein identifications. Peptide identifications were validated when established at a probability greater than 86,0 % to achieve an FDR less than 1,0 % using the Scaffold Local FalseDiscoveryRate (FDR) algorithm. Protein identifications were validated when established at a probability greater than 99,0 % probability to achieve an FDR less than 1,0 % and contained at least 2 identified peptides. Protein probabilities were assigned by the Protein Prophet algorithm [69].

### Bioinformatics tools

Datasets processed by Scaffold proteome software were submitted to the Contaminant Repository for Affinity Purification (CRAPome) and proteins identified were sorted by the fold change scores FC-A [24]. The STRING database was used to build protein–protein interaction networks [38]. The six types of interactions were included: Neighborhood in the Genome, Gene Fusion, Co-occurence across Genomes, Co-Expression, Experimental/Biochemical Data, Association in Curated Databases and Co-Mentioned in PubMed Abstracts. *In silico* analyses of gene expression was performed using Breast cancer data from Cancer Cell Line Encyclopedia (Broad Institute) and data are expressed in Log2 scale as Gene-centric Robust Multi-array Average (RMA)-normalized mRNA expression data using RMA Express Tool [70].

### Cell culture

The human mammary epithelial cells (MCF10A) were cultured in (1:1) DMEM:F12 medium supplemented with 5% horse serum, 100 ng/ml cholera toxin, 20 ng/ml EGF, 500 ng/ml hydrocortisone, and 10 μg/ml insulin. ADAM12L-overexpressing MCF10A cells were obtained by expressing GFP-ADAM12L C-terminal fusion protein in MCF10A cells using a lentiviral expression as previously described [11]. Breast cancer cell line HS578T and BT549, Cos7 and NIH3T3 cells were cultured in DMEM supplemented with 10% Fetal Bovine Serum (FBS). NIH3T3-Src cells were kindly provided by Frederic Saltel (Bordeaux Research in Translational Oncology, Bordeaux, France).

### Transfections

For siRNA transfections, cells were plated at 3 × 10^5^ cells per 60 mm dish the day before transfection. Transfections were performed using Lipofectamine RNAimax (Invitrogen^®^, Life Technologies) according to manufacturer instructions. Cells were used 72 hours post transfection. Sequences or references for siRNAs (Eurogentec) used are as follows: SiCtrl (ref: SR-CL000-005), si1ADAM12L CGACUGCUGUUUACAAAUAdTdT, si2A DAM12 GCAAAGAACUGAUCAUAAAdTdT, si3ADA M12 GGUGAUCCUUAUGGCAACUdTdT, si1ZO-1 GUU AUACGAGCGAUCUCAUdTdT, si2ZO-1 GGAGGAAAC AGCUAUAUGGdTdT, si3ZO-1 GACGAGAUAAUCC UCAUUUdTdT and si1PKCε GAGUGUAUGUGAUC AUCGAdTdT, si2PKCε CGUCAUCCUUCAGGAUG AUdTdT, si3PKCε GCAUCUUGAAAGCUUUCAUdTdT.

### Immunoprecipitation and western blotting

For immunoblotting, cell lysates were subjected to SDS-polyacrylamide gel electrophoresis (SDS-PAGE) and transferred to nitrocellulose membranes (GE Healthcare, UK). Blots were incubated for 30 min in Tris-buffered saline containing 0.1% Tween 20 and 5% non-fat dry milk and further incubated for two hours (or overnight at 4° C) with the following antibodies: rabbit anti-ADAM12 (Sigma), rabbit anti-ZO-1 (Life Technologies). The bound antibodies were detected with horseradish peroxidase-conjugated to either anti rabbit (Pierce) or anti-mouse (BioRad, Ivry, France) IgG or Protein A using an enhanced chemiluminescence system (Millipore, Billerica, MA, USA).

For immunoprecipitation, cell extracts were prepared in RIPA buffer and pre- incubated for 1 hour with sepharose-coupled protein-G beads (Amersham) alone to reduce non specific protein binding and then with sepharose-coupled protein G prebound with 1μg of either specific antibodies or control rabbit IgG. The beads were washed five times in buffer and samples were analyzed by sodium dodecyl sulfate-polyacrylamide gel electrophoresis and immunoblotting.

### Immunostaining

Cells were fixed with 4% paraformaldehyde for 15 min without permeabilization and washed twice with PBS. Then cells were blocked in 1% BSA, PBS for 1 hour before incubation with primary antibodies: rabbit anti-ADAM12 (generous gift from Dr Zolkiewska), mouse anti-ZO-1 (Life Technologies), rabbit or mouse anti-cortactin (Abcam), followed by Alexa Fluor 488-conjugated anti-rabbit (Cell Signaling) or by fluoprobes 647H-conjugated anti-mouse secondary antibodies (Interchim). The actin cytoskeleton is visualized by fluorescent phalloidin 547H (Interchim). Nuclei were stained with Hoechst 33258 dye (Invitrogen). The slides were washed, mounted in fluorescent mounting medium DAKO (Dako, North America, Inc) and viewed using an automated microscope.

### Gelatin matrix degradation assay

A matrix degradation assay was performed using fluorescent gelatin. Fluorescent 488 gelatin coverslips were prepared following manufacturer's instructions. In brief, glass coverslips were washed overnight in 10% HCl, and sterilized in ethanol. Coverslips were coated with 50 μg/ml poly-l-lysine in PBS (Sigma-Aldrich) and washed in PBS, then coated at room temperature for 20 min with 10:1 Oregon Green 488 conjugated gelatin (G13186, Life technologies) with 0.2% wt/vol unlabeled gelatin solution (G1393, Sigma) in PBS at room temperature, and incubated in 0.5% glutaraldehyde in PBS for 40 min. Gelatin matrix were then quenched with 5 mg/ml sodium borohydride and washed with PBS before seeding cells. HS578T cells were plated for 16 h in serum-containing media and coverslips were processed as described in the immunofluorescence section. Gelatin degradation resulted in black area depleted of fluorescent gelatin. These degraded area were quantified with the threshold tool of ImageJ software, and a degradation index was calculated as a ratio between the area of degraded gelatin and the cell area measured by Phalloidin 547H staining (F-Actin). At least 330 cells per conditions were analyzed. Images shown are from a single representative experiment out of a minimum of three repeats.

### Proximity ligation assay

The DUOLink PLA kit (Sigma) was used to detect protein–protein interactions. HS578T cells were cultured and transfected with siRNA (Ctrl, or ADAM12L, or ZO-1, or PKCε), fixed in 4% PFA for 15 min at room temperature then blocked with Duolink blocking buffer for 30 min at 37° C. Cells were incubated with primary antibodies diluted in Duolink antibody diluents for 2 h at room temperature, washed and then further incubated for another 1 hr at 37° C with species specific PLA probes (PLUS and MINUS probes) under hybridization conditions and in the presence of 2 additional oligonucleotides to facilitate hybridization of PLA probes only if they were in close proximity (<40 nm). A ligation mixture and ligase were then added to join the two hybridized oligonucleotides to form a closed circle. Several cycles of rolling-circle amplification using the ligated circle as a template were performed by adding an amplification solution to generate a concatemeric product extending from the oligonucleotide arm of the PLA probe. Lastly, a detection solution consisting of fluorescently labeled oligonucleotides was added, and the labeled oligonucleotides were hybridized to the concatemeric products. The signal was detected as a distinct fluorescent dot in the FITC green channel and analyzed by fluorescence microscopy. Specificity of PLA was determined by using negative controls consisting of samples treated as described but with only primary or secondary antibodies. PLA was quantified using Image J software. Dots were counted from 100 to 300 cells.

### Matrigel invasion assay

Matrigel invasion assay was performed using 24-well BD BioCoat™ Matrigel Invasion Chambers 8 μm pore size (BD Biosciences). 2 × 10^4^ HS578T cells were seeded in the upper chamber of a 12-well plate, coated with growth factor-reduced Matrigel (Corning), in 500 μl serum-free medium. The lower chamber was filled with 500 μl medium containing 10% FBS. The chamber was incubated at 37° C, for 16 h. At the end of incubation, cells in the upper surface of the membrane were removed with a cotton swab. Invading cells, to the lower surface of the membrane, were fixed with 4% PFA for 2 min at room temperature, washed twice with PBS, permeabilized with methanol 100% for 20 min at room temperature and stained with Giemsa for 15 min at room temperature in dark. The images were obtained using an inverted microscope (Zeiss) and the cells were counted in five different view fields, using NIH ImageJ software (Magnification 10×). Results are expressed as the mean ± SD of three independent experiments.

### Epifluorescence and confocal imaging

Gelatin degradation assays were imaged using a LEICA- DMRXA2 epifluorescence microscope with a 40× PL Fluotar (O.N.; 1.00) oil objective. Confocal imaging used a LEICA SP8 DMI 6000 CS microscope using oil immersion objectives (40×/1.30 HC PL APO) and (63×/1.4 HC PL APO). Images were acquired using the LAS-AF (Leica, Wetzlar, Germany) software. Z-stacks were acquired with Surplatine superZ galvo at a step size of 500 nm. Brightness and contrast adjustments of images were realized using the imageJ software.

### Statistical analyses

All data are presented as the mean ± SEM from n independent experiments. Significant differences between conditions were evaluated using the Wilcoxon test for matrix degradation analyses and using the Student's *t*-test for western blotting densitometry. All analyses were carried out using GraphPad Prism version 7.00 for Windows (GraphPad Software, La Jolla, CA,USA). ^*^P < 0.05, ^**^P < 0.01 and ^***^P < 0.001 were considered significant.

## SUPPLEMENTARY MATERIALS FIGURES AND TABLES







## Abbreviations

ADAM: a disintegrin and metalloprotease
EMT: epithelial mesenchymal transition
ECM: extracellular matrix
TGF-β: transforming growth factor-beta
siRNA: small interfering RNA

## References

[1] Nieto MA, Huang RYJ, Jackson RA, Thiery JP (2016). EMT: 2016. Cell.

[2] Lamouille S, Xu J, Derynck R (2014). Molecular mechanisms of epithelial-mesenchymal transition. Nat Rev Mol Cell Biol.

[3] Mochizuki S, Okada Y (2007). ADAMs in cancer cell proliferation and progression. Cancer Sci.

[4] Hamada S, Satoh K, Fujibuchi W, Hirota M, Kanno A, Unno J, Masamune A, Kikuta K, Kume K, Shimosegawa T (2012). MiR-126 acts as a tumor suppressor in pancreatic cancer cells via the regulation of ADAM9. Mol Cancer Res.

[5] Cai M, Wang Z, Zhang J, Zhou H, Jin L, Bai R, Weng Y (2015). Adam17, a Target of Mir-326, Promotes Emt-Induced Cells Invasion in Lung Adenocarcinoma. Cell Physiol Biochem.

[6] Xu M, Zhou H, Zhang C, He J, Wei H, Zhou M, Lu Y, Sun Y, Ding JW, Zeng J, Peng W, Du F, Gong A (2016). ADAM17 promotes epithelial-mesenchymal transition via TGF-β/Smad pathway in gastric carcinoma cells. Int J Oncol.

[7] Kveiborg M, Fröhlich C, Albrechtsen R, Tischler V, Dietrich N, Holck P, Kronqvist P, Rank F, Mercurio AM, Wewer UM (2005). A role for ADAM12 in breast tumor progression and stromal cell apoptosis. Cancer Res.

[8] Roy R, Rodig S, Bielenberg D, Zurakowski D, Moses MA (2011). ADAM12 transmembrane and secreted isoforms promote breast tumor growth: a distinct role for ADAM12-S protein in tumor metastasis. J Biol Chem.

[9] Li H, Duhachek-Muggy S, Qi Y, Hong Y, Behbod F, Zolkiewska A (2012). An essential role of metalloprotease-disintegrin ADAM12 in triple-negative breast cancer. Breast Cancer Res Treat.

[10] Li H, Duhachek-Muggy S, Dubnicka S, Zolkiewska A (2013). Metalloproteinase-disintegrin ADAM12 is associated with a breast tumor-initiating cell phenotype. Breast Cancer Res Treat.

[11] Ruff M, Leyme A, Le Cann F, Bonnier D, Le Seyec J, Chesnel F, Fattet L, Rimokh R, Baffet G, Théret N (2015). The Disintegrin and Metalloprotease ADAM12 Is Associated with TGF-β-Induced Epithelial to Mesenchymal Transition. PLoS One.

[12] Eto K, Puzon-McLaughlin W, Sheppard D, Sehara-Fujisawa A, Zhang XP, Takada Y (2000). RGD-independent binding of integrin alpha9beta1 to the ADAM-12 and −15 disintegrin domains mediates cell-cell interaction. J Biol Chem.

[13] Kawaguchi N, Sundberg C, Kveiborg M, Moghadaszadeh B, Asmar M, Dietrich N, Thodeti CK, Nielsen FC, Möller P, Mercurio AM, Albrechtsen R, Wewer UM (2003). ADAM12 induces actin cytoskeleton and extracellular matrix reorganization during early adipocyte differentiation by regulating beta1 integrin function. J Cell Sci.

[14] Iba K, Albrechtsen R, Gilpin B, Fröhlich C, Loechel F, Zolkiewska A, Ishiguro K, Kojima T, Liu W, Langford JK, Sanderson RD, Brakebusch C, Fässler R (2000). The cysteine-rich domain of human ADAM 12 supports cell adhesion through syndecans and triggers signaling events that lead to beta1 integrin-dependent cell spreading. J Cell Biol.

[15] Atfi A, Dumont E, Colland F, Bonnier D, L’Helgoualc'h A, Prunier C, Ferrand N, Clément B, Wewer UM, Théret N (2007). The disintegrin and metalloproteinase ADAM12 contributes to TGF-beta signaling through interaction with the type II receptor. J Cell Biol.

[16] Suzuki A, Kadota N, Hara T, Nakagami Y, Izumi T, Takenawa T, Sabe H, Endo T (2000). Meltrin alpha cytoplasmic domain interacts with SH3 domains of Src and Grb2 and is phosphorylated by v-Src. Oncogene.

[17] Abram CL, Seals DF, Pass I, Salinsky D, Maurer L, Roth TM, Courtneidge SA (2003). The adaptor protein fish associates with members of the ADAMs family and localizes to podosomes of Src-transformed cells. J Biol Chem.

[18] Asakura M, Kitakaze M, Takashima S, Liao Y, Ishikura F, Yoshinaka T, Ohmoto H, Node K, Yoshino K, Ishiguro H, Asanuma H, Sanada S, Matsumura Y (2002). Cardiac hypertrophy is inhibited by antagonism of ADAM12 processing of HB-EGF: metalloproteinase inhibitors as a new therapy. Nat Med.

[19] Sundberg C, Thodeti CK, Kveiborg M, Larsson C, Parker P, Albrechtsen R, Wewer UM (2004). Regulation of ADAM12 cell-surface expression by protein kinase C epsilon. J Biol Chem.

[20] Bourd-Boittin K, Le Pabic H, Bonnier D, L’Helgoualc'h A, Théret N (2008). RACK1, a new ADAM12 interacting protein. Contribution to liver fibrogenesis. J Biol Chem.

[21] Leyme A, Bourd-Boittin K, Bonnier D, Falconer A, Arlot-Bonnemains Y, Théret N (2012). Identification of ILK as a new partner of the ADAM12 disintegrin and metalloprotease in cell adhesion and survival. Mol Biol Cell.

[22] Galliano MF, Huet C, Frygelius J, Polgren A, Wewer UM, Engvall E (2000). Binding of ADAM12, a marker of skeletal muscle regeneration, to the muscle-specific actin-binding protein, alpha -actinin-2, is required for myoblast fusion. J Biol Chem.

[23] Mori S, Tanaka M, Nanba D, Nishiwaki E, Ishiguro H, Higashiyama S, Matsuura N (2003). PACSIN3 binds ADAM12/meltrin alpha and up-regulates ectodomain shedding of heparin-binding epidermal growth factor-like growth factor. J Biol Chem.

[24] Mellacheruvu D, Wright Z, Couzens AL, Lambert JP, St-Denis NA, Li T, Miteva YV, Hauri S, Sardiu ME, Low TY, Halim VA, Bagshaw RD, Hubner NC (2013). The CRAPome: a contaminant repository for affinity purification-mass spectrometry data. Nat Methods.

[25] King SJ, Nowak K, Suryavanshi N, Holt I, Shanahan CM, Ridley AJ (2014). Nesprin-1 and nesprin-2 regulate endothelial cell shape and migration. Cytoskeleton (Hoboken).

[26] Saafan H, Foerster S, Parra-Guillen ZP, Hammer E, Michaelis M, Cinatl J, Völker U, Fröhlich H, Kloft C, Ritter CA (2016). Utilising the EGFR interactome to identify mechanisms of drug resistance in non-small cell lung cancer - Proof of concept towards a systems pharmacology approach. Eur J Pharm Sci.

[27] Sasahira T, Kirita T, Nishiguchi Y, Kurihara M, Nakashima C, Bosserhoff AK, Kuniyasu H (2016). A comprehensive expression analysis of the MIA gene family in malignancies: MIA gene family members are novel, useful markers of esophageal, lung, and cervical squamous cell carcinoma. Oncotarget.

[28] Jilaveanu LB, Parisi F, Barr ML, Zito CR, Cruz-Munoz W, Kerbel RS, Rimm DL, Bosenberg MW, Halaban R, Kluger Y, Kluger HM (2015). PLEKHA5 as a Biomarker and Potential Mediator of Melanoma Brain Metastasis. Clin Cancer Res.

[29] Mound A, Rodat-Despoix L, Bougarn S, Ouadid-Ahidouch H, Matifat F (2013). Molecular interaction and functional coupling between type 3 inositol 1,4,5-trisphosphate receptor and BKCa channel stimulate breast cancer cell proliferation. Eur J Cancer.

[30] Wilson NR, Olm-Shipman AJ, Acevedo DS, Palaniyandi K, Hall EG, Kosa E, Stumpff KM, Smith GJ, Pitstick L, Liao EC, Bjork BC, Czirok A, Saadi I (2016). SPECC1L deficiency results in increased adherens junction stability and reduced cranial neural crest cell delamination. Sci Rep.

[31] Runkle EA, Mu D (2013). Tight junction proteins: from barrier to tumorigenesis. Cancer Lett.

[32] Jauliac S, López-Rodriguez C, Shaw LM, Brown LF, Rao A, Toker A (2002). The role of NFAT transcription factors in integrin-mediated carcinoma invasion. Nat Cell Biol.

[33] Feldman DE, Chen C, Punj V, Machida K (2013). The TBC1D15 oncoprotein controls stem cell self-renewal through destabilization of the Numb-p53 complex. PLoS One.

[34] Ruiz-Lafuente N, Alcaraz-García MJ, García-Serna AM, Sebastián-Ruiz S, Moya-Quiles MR, García-Alonso AM, Parrado A (2015). Dock10, a Cdc42 and Rac1 GEF, induces loss of elongation, filopodia, and ruffles in cervical cancer epithelial HeLa cells. Biol Open.

[35] Gadea G, Sanz-Moreno V, Self A, Godi A, Marshall CJ (2008). DOCK10-mediated Cdc42 activation is necessary for amoeboid invasion of melanoma cells. Curr Biol.

[36] Li J, Tan Q, Yan M, Liu L, Lin H, Zhao F, Bao G, Kong H, Ge C, Zhang F, Yu T, Li J, He X (2014). miRNA-200c inhibits invasion and metastasis of human non-small cell lung cancer by directly targeting ubiquitin specific peptidase 25. Mol Cancer.

[37] Aghababaei M, Perdu S, Irvine K, Beristain AG (2014). A disintegrin and metalloproteinase 12 (ADAM12) localizes to invasive trophoblast, promotes cell invasion and directs column outgrowth in early placental development. Mol Hum Reprod.

[38] Szklarczyk D, Morris JH, Cook H, Kuhn M, Wyder S, Simonovic M, Santos A, Doncheva NT, Roth A, Bork P, Jensen LJ, von Mering C (2017). The STRING database in 2017: quality-controlled protein-protein association networks, made broadly accessible. Nucleic Acids Res.

[39] Kang Q, Cao Y, Zolkiewska A (2001). Direct interaction between the cytoplasmic tail of ADAM 12 and the Src homology 3 domain of p85alpha activates phosphatidylinositol 3-kinase in C2C12 cells. J Biol Chem.

[40] Reichert M, Müller T, Hunziker W (2000). The PDZ domains of zonula occludens-1 induce an epithelial to mesenchymal transition of Madin-Darby canine kidney I cells. Evidence for a role of beta-catenin/Tcf/Lef signaling. J Biol Chem.

[41] Hougaard S, Loechel F, Xu X, Tajima R, Albrechtsen R, Wewer UM (2000). Trafficking of human ADAM 12-L: retention in the trans-Golgi network. Biochem Biophys Res Commun.

[42] Barretina J, Caponigro G, Stransky N, Venkatesan K, Margolin AA, Kim S, Wilson CJ, Lehár J, Kryukov GV, Sonkin D, Reddy A, Liu M, Murray L (2012). The Cancer Cell Line Encyclopedia enables predictive modelling of anticancer drug sensitivity. Nature.

[43] Neve RM, Chin K, Fridlyand J, Yeh J, Baehner FL, Fevr T, Clark L, Bayani N, Coppe JP, Tong F, Speed T, Spellman PT, DeVries S (2006). A collection of breast cancer cell lines for the study of functionally distinct cancer subtypes. Cancer Cell.

[44] Albrechtsen R, Stautz D, Sanjay A, Kveiborg M, Wewer UM (2011). Extracellular engagement of ADAM12 induces clusters of invadopodia with localized ectodomain shedding activity. Exp Cell Res.

[45] Hirakawa H, Shibata K, Nakayama T (2009). Localization of cortactin is associated with colorectal cancer development. Int J Oncol.

[46] Murphy DA, Courtneidge SA (2011). The “ins” and “outs” of podosomes and invadopodia: characteristics, formation and function. Nat Rev Mol Cell Biol.

[47] Polette M, Gilles C, Nawrocki-Raby B, Lohi J, Hunziker W, Foidart JM, Birembaut P (2005). Membrane-type 1 matrix metalloproteinase expression is regulated by zonula occludens-1 in human breast cancer cells. Cancer Res.

[48] Kremerskothen J, Stölting M, Wiesner C, Korb-Pap A, van Vliet V, Linder S, Huber TB, Rottiers P, Reuzeau E, Genot E, Pavenstädt H (2011). Zona occludens proteins modulate podosome formation and function. FASEB J.

[49] Albrechtsen R, Kveiborg M, Stautz D, Vikeså J, Noer JB, Kotzsh A, Nielsen FC, Wewer UM, Fröhlich C (2013). ADAM12 redistributes and activates MMP-14, resulting in gelatin degradation, reduced apoptosis and increased tumor growth. J Cell Sci.

[50] Tuomi S, Mai A, Nevo J, Laine JO, Vilkki V, Ohman TJ, Gahmberg CG, Parker PJ, Ivaska J (2009). PKCepsilon regulation of an alpha5 integrin-ZO-1 complex controls lamellae formation in migrating cancer cells. Sci Signal.

[51] Yagami-Hiromasa T, Sato T, Kurisaki T, Kamijo K, Nabeshima Y, Fujisawa-Sehara A (1995). A metalloprotease-disintegrin participating in myoblast fusion. Nature.

[52] Okada A, Mochizuki S, Yatabe T, Kimura T, Shiomi T, Fujita Y, Matsumoto H, Sehara-Fujisawa A, Iwamoto Y, Okada Y (2008). ADAM-12 (meltrin alpha) is involved in chondrocyte proliferation via cleavage of insulin-like growth factor binding protein 5 in osteoarthritic cartilage. Arthritis Rheum.

[53] Masaki M, Kurisaki T, Shirakawa K, Sehara-Fujisawa A (2005). Role of meltrin {alpha} (ADAM12) in obesity induced by high- fat diet. Endocrinology.

[54] Cao Y, Zhao Z, Gruszczynska-Biegala J, Zolkiewska A (2003). Role of metalloprotease disintegrin ADAM12 in determination of quiescent reserve cells during myogenic differentiation *in vitro*. Mol Cell Biol.

[55] Inoue D, Reid M, Lum L, Krätzschmar J, Weskamp G, Myung YM, Baron R, Blobel CP (1998). Cloning and initial characterization of mouse meltrin beta and analysis of the expression of four metalloprotease-disintegrins in bone cells. J Biol Chem.

[56] Yilmaz M, Christofori G (2009). EMT, the cytoskeleton, and cancer cell invasion. Cancer Metastasis Rev.

[57] Fanning AS, Anderson JM (2009). Zonula occludens-1 and −2 are cytosolic scaffolds that regulate the assembly of cellular junctions. Ann N Y Acad Sci.

[58] Katsuno T, Umeda K, Matsui T, Hata M, Tamura A, Itoh M, Takeuchi K, Fujimori T, Nabeshima Y, Noda T, Tsukita S, Tsukita S (2008). Deficiency of zonula occludens-1 causes embryonic lethal phenotype associated with defected yolk sac angiogenesis and apoptosis of embryonic cells. Mol Biol Cell.

[59] Xu J, Kausalya PJ, Phua DCY, Ali SM, Hossain Z, Hunziker W (2008). Early embryonic lethality of mice lacking ZO-2, but Not ZO-3, reveals critical and nonredundant roles for individual zonula occludens proteins in mammalian development. Mol Cell Biol.

[60] Umeda K, Ikenouchi J, Katahira-Tayama S, Furuse K, Sasaki H, Nakayama M, Matsui T, Tsukita S, Furuse M, Tsukita S (2006). ZO-1 and ZO-2 independently determine where claudins are polymerized in tight-junction strand formation. Cell.

[61] Huang RYJ, Guilford P, Thiery JP (2012). Early events in cell adhesion and polarity during epithelial-mesenchymal transition. J Cell Sci.

[62] Smalley KSM, Brafford P, Haass NK, Brandner JM, Brown E, Herlyn M (2005). Up-regulated expression of zonula occludens protein-1 in human melanoma associates with N-cadherin and contributes to invasion and adhesion. Am J Pathol.

[63] Taliana L, Benezra M, Greenberg RS, Masur SK, Bernstein AM (2005). ZO-1: lamellipodial localization in a corneal fibroblast wound model. Invest Ophthalmol Vis Sci.

[64] Díaz B, Yuen A, Iizuka S, Higashiyama S, Courtneidge SA (2013). Notch increases the shedding of HB-EGF by ADAM12 to potentiate invadopodia formation in hypoxia. J Cell Biol.

[65] Eckert MA, Santiago-Medina M, Lwin TM, Kim J, Courtneidge SA, Yang J (2017). ADAM12 induction by Twist1 promotes tumor invasion and metastasis via regulation of invadopodia and focal adhesions. J Cell Sci.

[66] Stengel K, Zheng Y (2011). Cdc42 in oncogenic transformation, invasion, and tumorigenesis. Cell Signal.

[67] Huo L, Wen W, Wang R, Kam C, Xia J, Feng W, Zhang M (2011). Cdc42-dependent formation of the ZO-1/MRCKβ complex at the leading edge controls cell migration. EMBO J.

[68] Akita Y (2008). Protein kinase Cepsilon: multiple roles in the function of, and signaling mediated by, the cytoskeleton. FEBS J.

[69] Nesvizhskii AI, Keller A, Kolker E, Aebersold R (2003). A statistical model for identifying proteins by tandem mass spectrometry. Anal Chem.

[70] Irizarry RA, Hobbs B, Collin F, Beazer-Barclay YD, Antonellis KJ, Scherf U, Speed TP (2003). Exploration, normalization, and summaries of high density oligonucleotide array probe level data. Biostatistics.

[71] Galliher AJ, Schiemann WP (2007). Src phosphorylates Tyr284 in TGF-beta type II receptor and regulates TGF-beta stimulation of p38 MAPK during breast cancer cell proliferation and invasion. Cancer Res.

