# Correction: Proteomic screening identifies the zonula occludens protein ZO-1 as a new partner for ADAM12 in invadopodia-like structures

**DOI:** 10.18632/oncotarget.26339

**Published:** 2018-11-06

**Authors:** Bassil Dekky, Michael Ruff, Dominique Bonnier, Vincent Legagneux, Nathalie Théret

**Affiliations:** ^1^ Univ Rennes, Inserm, EHESP, Irset (Institut de recherche en santé, environnement et travail)-UMR_S1085, Rennes, France; ^2^ Present address: Institute of Biology Valrose (iBV) CNRS UMR7277 - INSERM U1091 – UNS University Nice Sophia Antipolis, Nice, France

**This article has been corrected:** The following sentence has been added to the first paragraph in the Material and Methods section: ‘Protein digestion and mass spectrometry analyses were performed by the Proteomics Platform of the CHU de Québec Research Center (Quebec, Qc,Canada).’

**Proteomics analyses**

Immunoprecipitation of proteins associated with ADAM12L were performed using extracts from ADAM12L-overexpressing MCF10A cells and immunoprecipitation using Rabbit IgG was included as control. Proteins from immunoprecipitates were denaturated and size-separated by sodium dodecyl sulfate-polyacrylamide gel electrophoresis (SDS-PAGE). After cutting gel tracks in 10 bands, in-gel digestion of proteins was performed with trypsin and resulting peptides were injected into a capillary HPLC system coupled to a mass spectrometer via a nanospray ionization source (ES MS/MS). All MS/MS samples were analyzed using Mascot (Matrix Science, London, UK; version 2.4.1) and X! Tandem softwares. Mascot was set up to search the TAX_HomoSapiens_9606_20141128 database. X! Tandem was set up to search a subset of the TAX_HomoSapiens_9606_20141128 database. For protein identification, Scaffold software (version 4.4.1.1, Proteome Software Inc., Portland, OR) was used to validate MS/MS based peptide and protein identifications. Peptide identifications were validated when established at a probability greater than 86,0 % to achieve an FDR less than 1,0 % using the Scaffold Local FalseDiscoveryRate (FDR) algorithm. Protein identifications were validated when established at a probability greater than 99,0 % probability to achieve an FDR less than 1,0 % and contained at least 2 identified peptides. Protein probabilities were assigned by the Protein Prophet algorithm [69]. Protein digestion and mass spectrometry analyses were performed by the Proteomics Platform of the CHU de Québec Research Center (Quebec, Qc,Canada).

Original article: Oncotarget. 2018; 9:21366-21382. https://doi.org/10.18632/oncotarget.25106

