# Supplementary material for: Proteomic screening identifies the zonula occludens protein ZO-1 as a new partner for ADAM12 in invadopodia-like structures
Source: Oncotarget. 2018 Apr 20;9(30):21366–82. doi: 10.18632/oncotarget.25106 (PMC5940405; doi:10.18632/oncotarget.25106)
Supplement: Supplementary file 1 [file oncotarget-09-21366-s001.pdf]

Proteomic screening identifies the zonula occludens protein ZO-1 as a new partner for ADAM12 in invadopodia-like structures

SUPPLEMENTARY MATERIALS

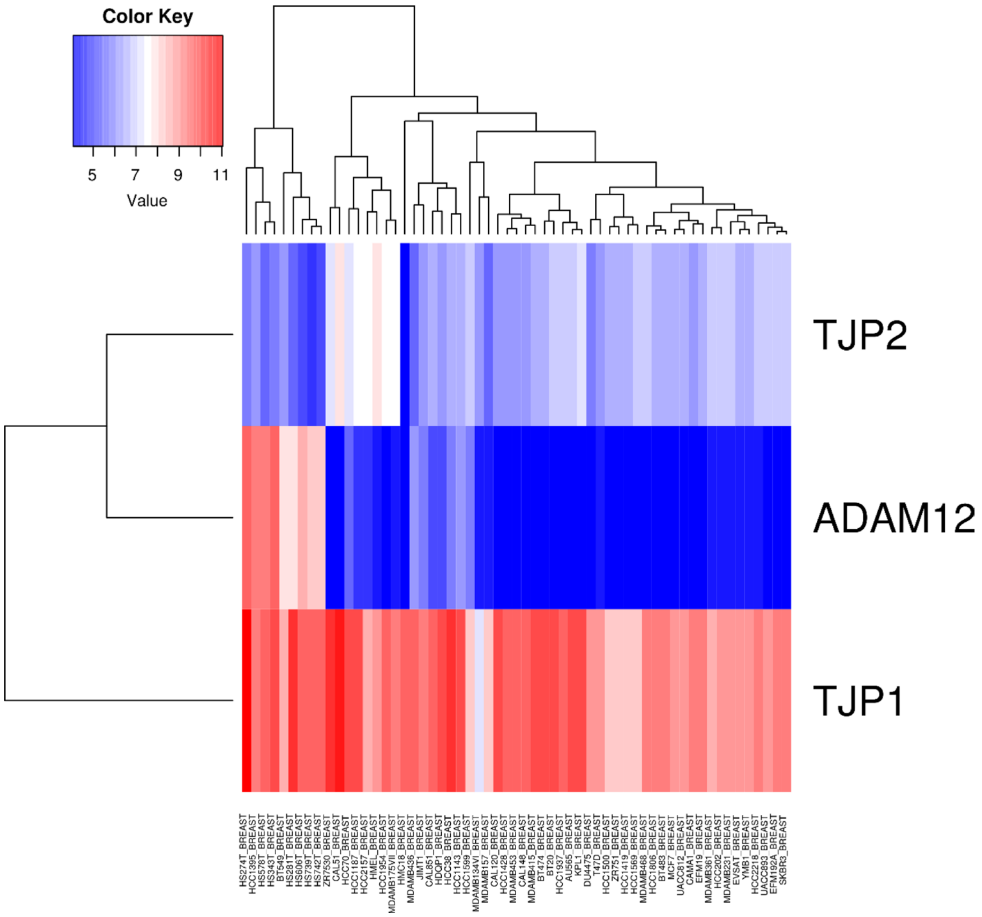

Supplementary Figure 1: Clustering analysis of ADAM12L and TJP1 (ZO-1) and TJP2 (ZO-2) gene expression in a panel of 59 breast cancer cell lines. Data are expressed in Log2 scale as Gene-centric RMA-normalized mRNA expression data.

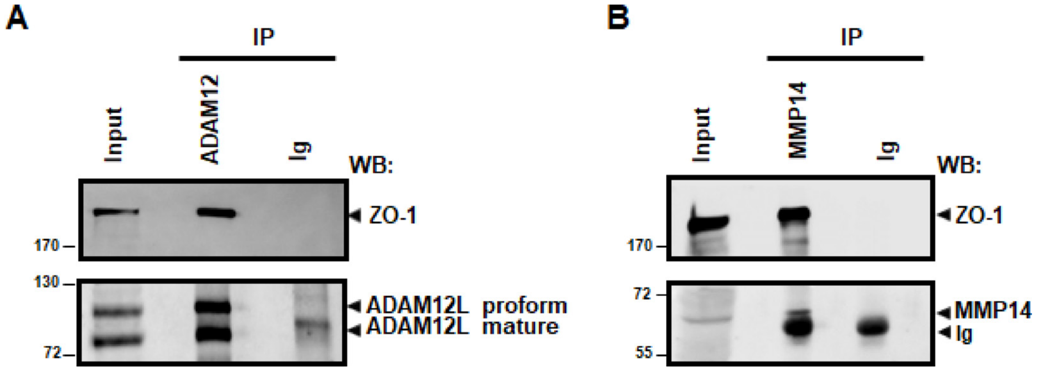

Supplementary Figure 2: Interaction of ZO-1 with ADAM12L and MMP14 in breast cancer cell line BT549. Crude extracts from BT549 cells were immunoprecipited (IP) with (**A**) anti-ADAM12 or (**B**) anti-MMP14 antibodies or control IgG (Ig) and immunoblotted with indicated antibodies (WB).

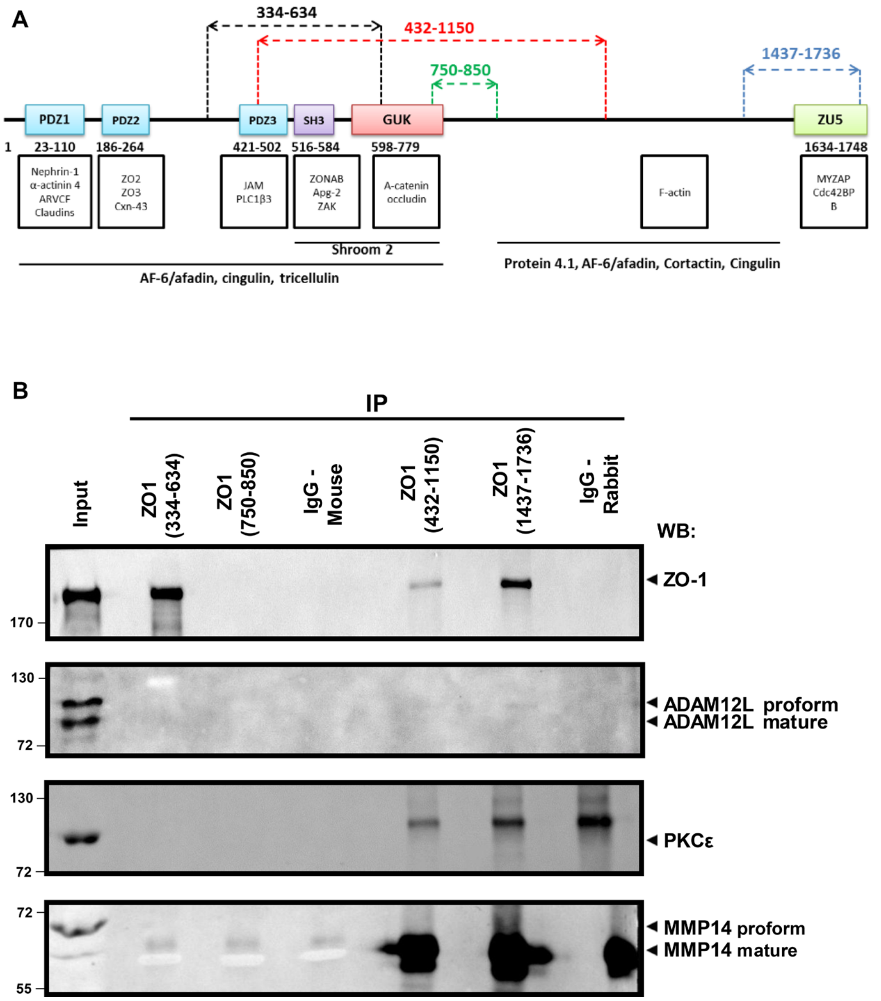

Supplementary Figure 3: (**A**) Schematic representation of ZO-1 domains recognized by commercial anti-ZO1 antibodies. The published interactions with several proteins are localized along the domains. (**B**) immunoprecipitation (IP) of ZO-1 from Hs578T cells using four different antibodies. Neither ADAM12, nor PKCε and MMP14 are detected.

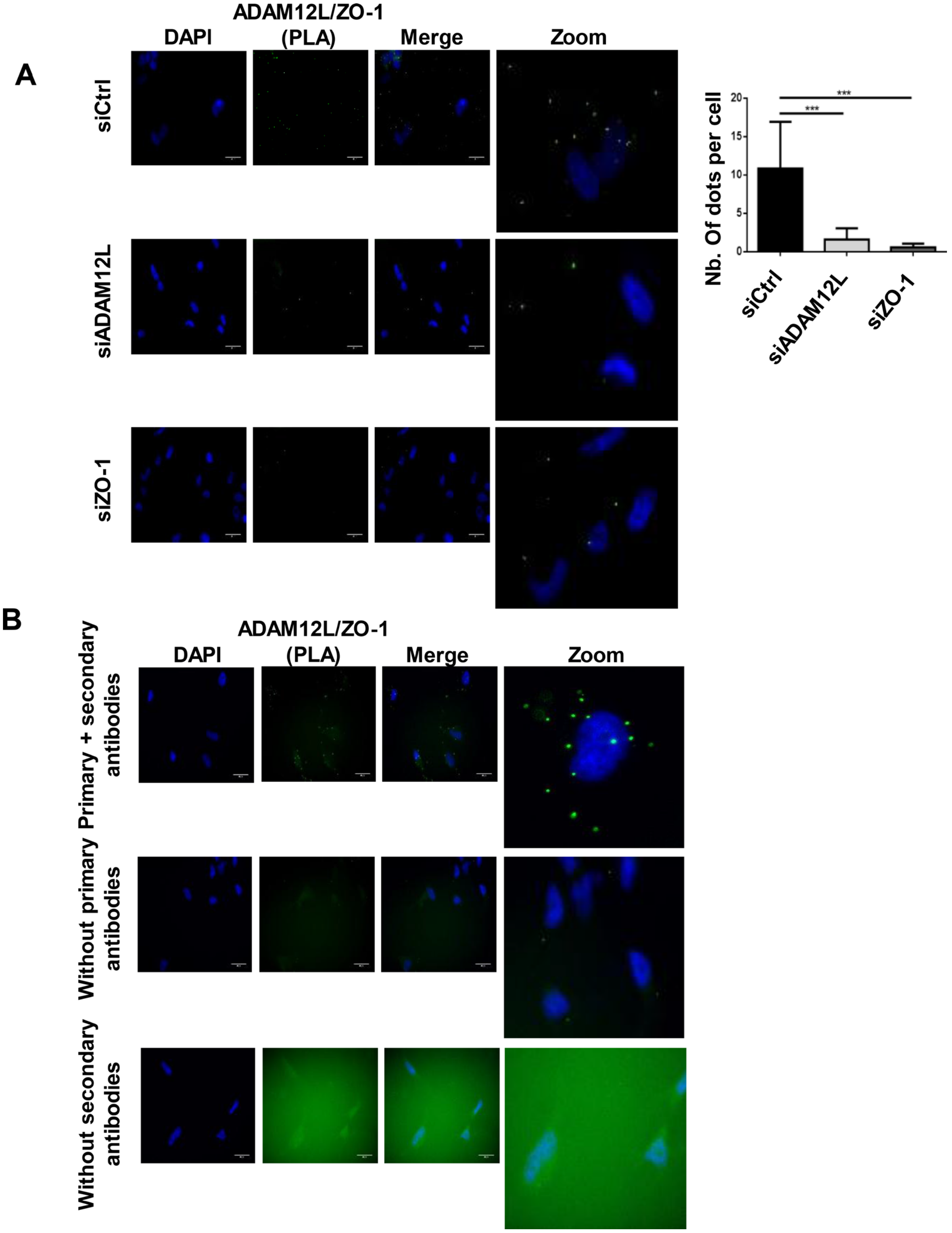

Supplementary Figure 4: ADAM12L interacts and co-localizes with ZO-1 in breast cancer cell line Hs578T. (**A**) Interaction between ZO-1 and ADAM12L was analyzed by proximity ligation assay in Hs578T cells silenced or not with either ADAM12L or ZO-1. Interaction results in fluorescent dots (green). Results are expressed as the mean ± SD of three independent experiments. (**B**) Negative controls are samples with only primary or secondary antibodies. Representative fields are shown.

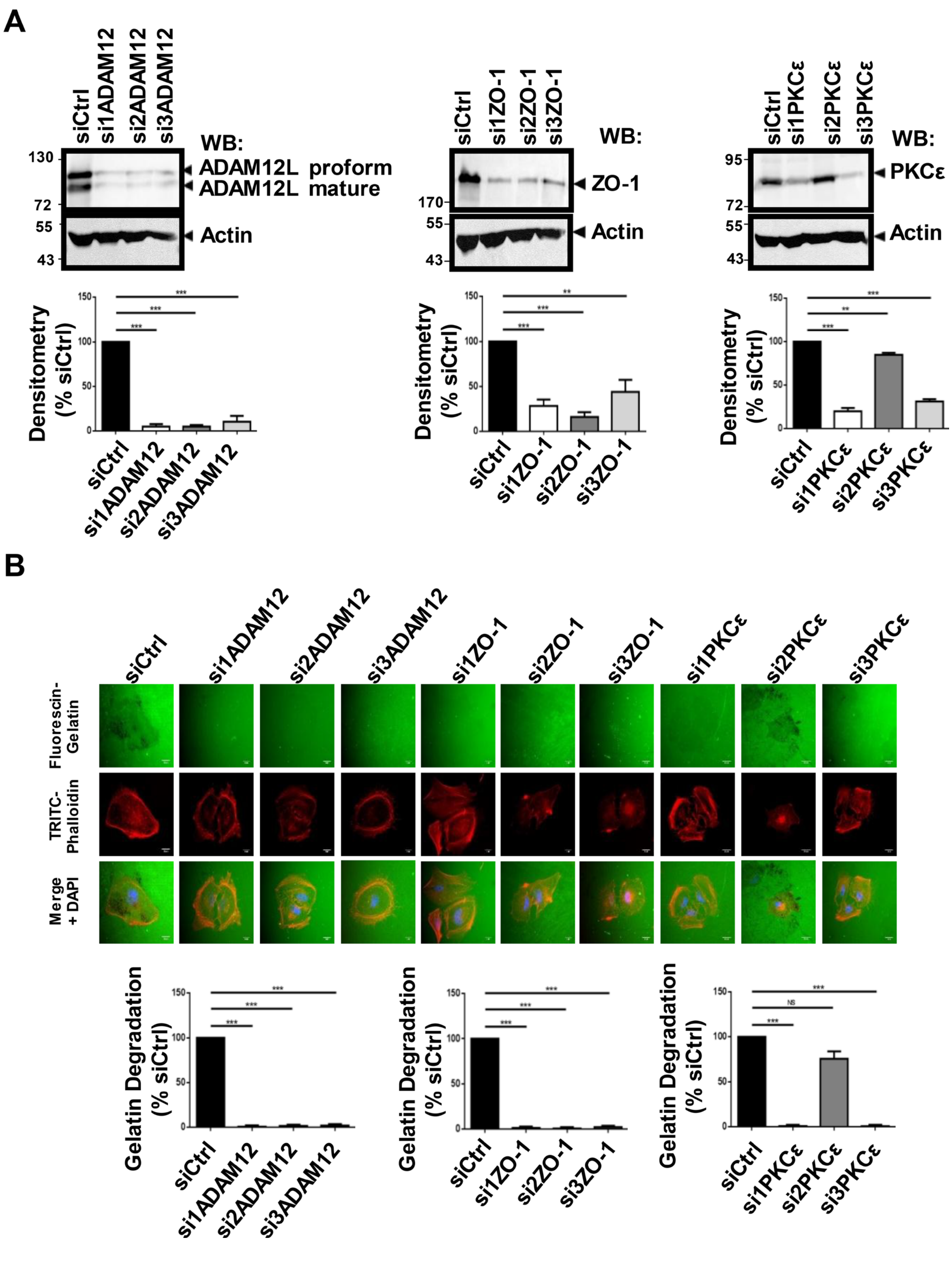

Supplementary Figure 5: Comparative analysis of three independent siRNA targeting ADAM12L, ZO-1 and PKCε. (**A**) Western blot analyses. (**B**) Gelatin degradation activity.

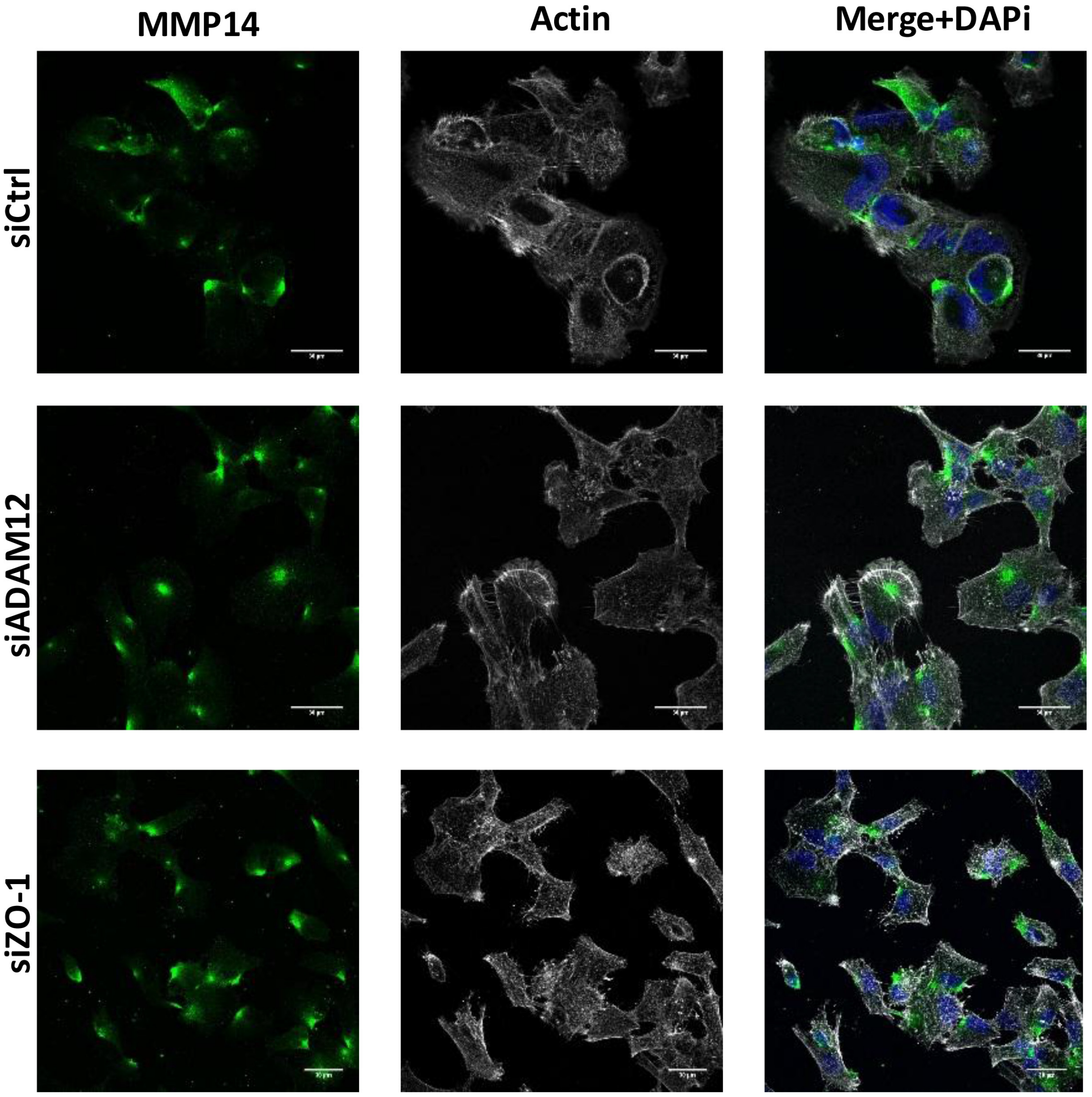

Supplementary Figure 6: Silencing ADAM12 and ZO-1 did not modify MMP14 localization. Hs578T cells were transfected with 2 nM non-targeting siRNA (siCtrl) or ADAM12L siRNA (siADAM12) or ZO-1 siRNA (SiZO-1). Cells were immunostained with antibodies against MMP14 (green). The actin cytoskeleton was stained by fluorescent phalloidin 547H (gray) and nuclei were stained with Hoechst 33258 dye (blue). Representative fields are shown.

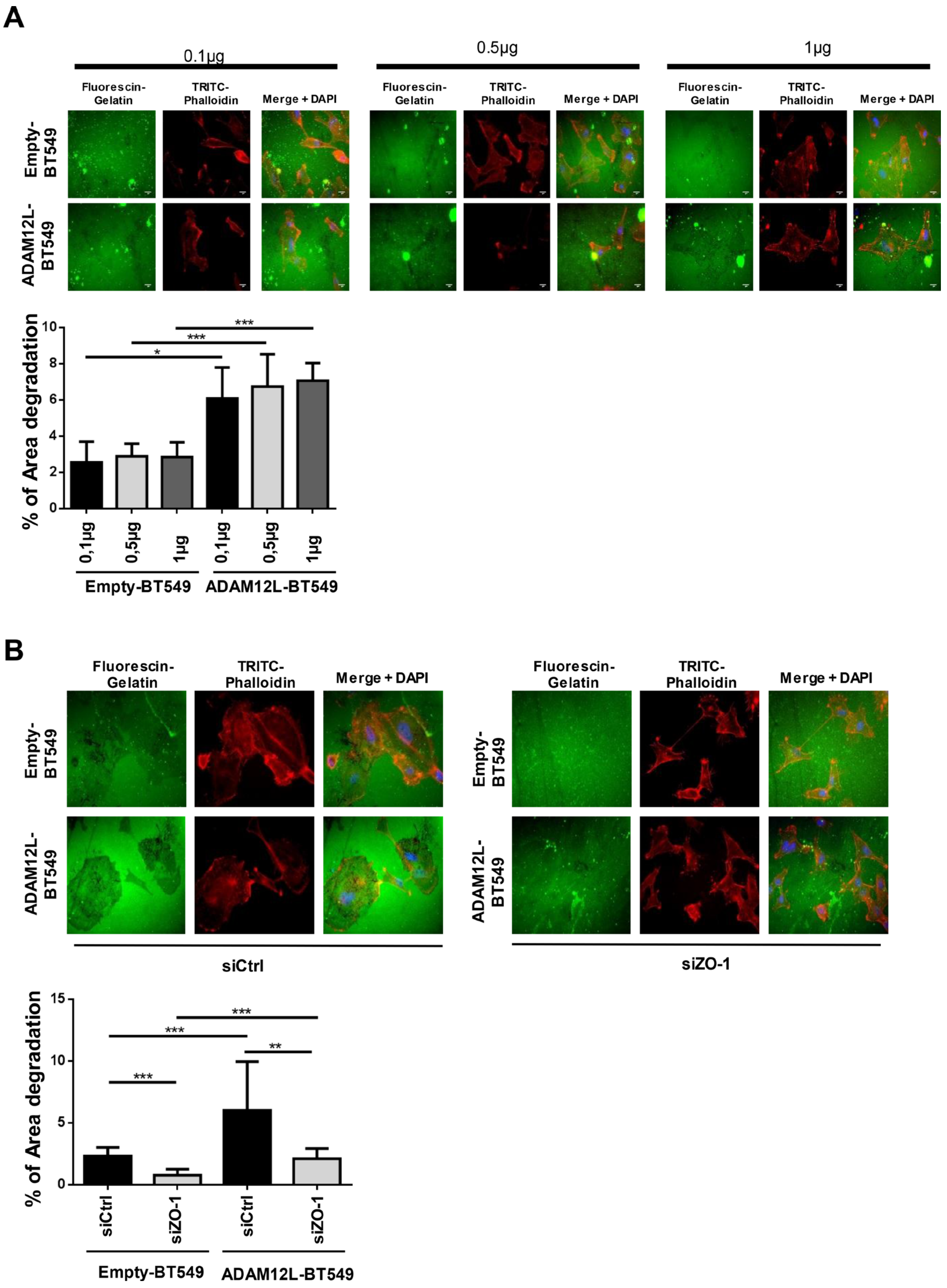

Supplementary Figure 7: Effect of silencing ZO-1 on ADAM12-dependent degradation activity. BT549 cells were transfected with ADAM12 expressing vector or empty vector as control and degradation activity was analyzed by using fluorescent gelatin 48 h post-transfection. (**A**) Effect of increase concentration of ADAM12 expressing vector. (**B**) Transfected cells with 0.5 µg of ADAM12 expressing vector were further treated with non-targeting siRNA (siCtrl) or ZO-1 siRNA (SiZO-1). One representative picture is shown for each condition (Actin in red, Gelatin in green). Results are expressed as the mean ± SD of three independent experiments in A and of 100 cell pictures in B ($^{***}P < 0.0001$).

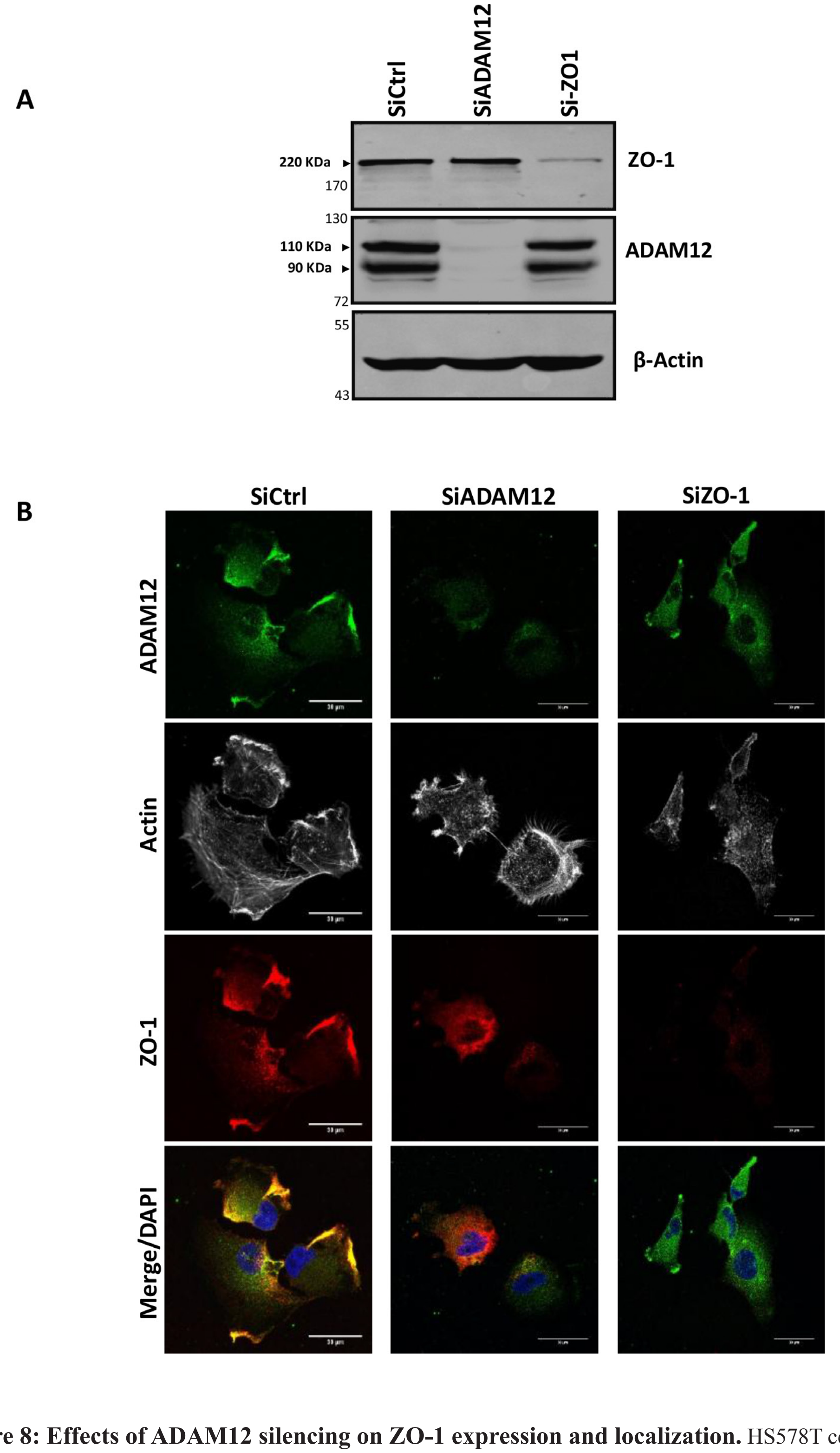

Supplementary Figure 8: Effects of ADAM12 silencing on ZO-1 expression and localization. HS578T cells were transfected with 2 nM non-targeting siRNA (SiCtrl) or ADAM12L siRNA (siADAM12) or ZO-1 siRNA (SiZO-1). (**A**) After 72 h, western blotting were used to confirm the efficiency and specificity of RNA interference in cell extracts. (**B**) Non permeabilized cells were immunostained with antibodies against ADAM12 (green) and ZO-1 (red). Representative fields are shown. Co-localization results in yellow cellular staining. The actin cytoskeleton was stained by fluorescent phalloidin 547H (gray) and nuclei were stained with Hoechst 33258 dye (blue). Decrease in staining of ADAM12 and ZO-1 was observed in silenced ADAM12L- and ZO-1- cells, respectively. Silencing ADAM12L led to decrease of ZO-1 signal in actin-rich structure while silencing ZO-1 did not induce significant changes in ADAM12 localization.

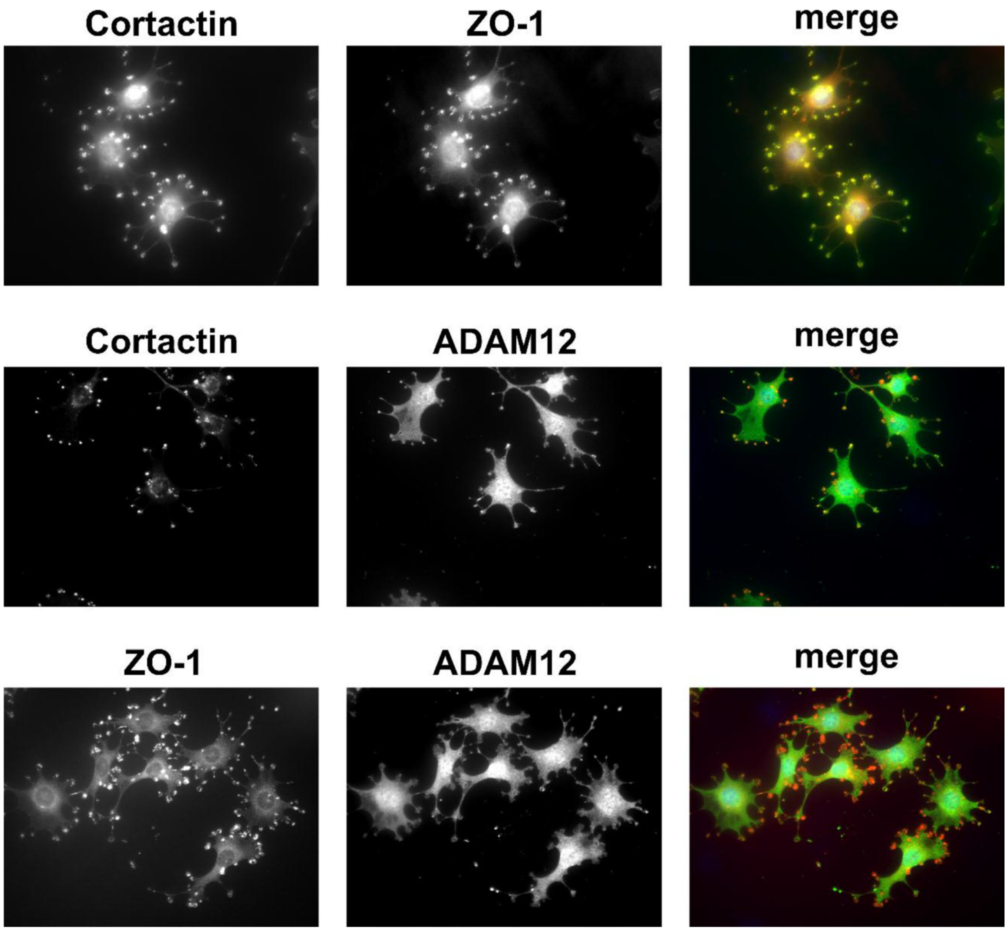

Supplementary Figure 9: ADAM12 and ZO-1 colocalized in podosomes of Src-transformed NIH3T3 cells. Cells were immunostained with antibodies against ADAM12 (green), ZO-1 (red) and cortactin (red). Single channel pictures are in gray scale and co-localization of ADAM12 and ZO-1 results in yellow cellular staining. Representative fields are shown.

Supplementary Table 1: List of protein specifically identified in antiADAM12 immunoprecipitates. See Supplementary_Table_1

Supplementary Table 2: List of proteins previously identified as ADAM12L interacting proteins

Gene symbol	Description	Reference
ACTN2	Actinin, alpha 2	Galliano *et al*, 2000
ACTN1	Actinin, alpha 1	Cao *et al*, 2001
GRB2	Growth factor receptor-bound protein 2	Suzuki *et al*, 2000
SRC	V-src sarcoma (Schmidt-Ruppin A-2) viral oncogene homolog (avian)	Suzuki *et al*, 2000; Kang *et al*, 2000
YES1	V-yes-1 Yamaguchi sarcoma viral oncogene homolog 1	Suzuki *et al*, 2000
PIK3R1	Phosphoinositide-3-kinase, regulatory subunit 1 (alpha)	Kang *et al*, 2001
PRKCE	Protein kinase C, epsilon	Sundberg *et al*, 2004
PRKCD	Protein kinase C, delta	Asakura *et al*, 2002
ITGB1	Integrin, beta 1 (fibronectin receptor, beta polypeptide, antigen CD29)	Kawaguchi *et al*, 2003
SH3PXD2A	SH3 and PX domains 2A	Abram *et al* , 2003
PACSIN3	Protein kinase C and casein kinase substrate in neurons 3	Mori *et al*, 2003
SH3D19	SH3 domain containing 19	Tanaka *et al*, 2004
TGFBR2	Transforming growth factor, beta receptor II (70/80 kDa)	Atfi *et al*, 2007
GNB2L1	Guanine nucleotide binding protein (G protein), beta polypeptide 2-like 1	Bourd-Boittin *et al*, 2008
ILK	Integrin-linked kinase	Leyme *et al*, 2012

Supplementary Table 3: Description of the types of annotation reported in STRING database

Annotation STRING	Neighborhood in the Genome:	Gene Fusions:	Cooccurence Across Genomes:	Co-Expression:	Experimental/ Biochemical Data:	Association in Curated Databases:	Co-Mentioned in PubMed Abstracts:	Combined Score:	Experimental/ Biochemical Data:	PMID
**Protein-protein interaction**										
IPTR3-SRC							yes (score 0.877). In addition, putative homologs are mentioned together in other species (score 0.073).	0.881		
IPTR3-GRB2							yes (score 0.846).	0.846		
TJP2-YES							yes (score 0.348). In addition, putative homologs are mentioned together in other species (score 0.218).	0.468		
TJPE2-SRC							yes (score 0.506). In addition, putative homologs are mentioned together in other species (score 0.218).	0.597		
TJP1-PRKCD						yes (score 0.800).	yes (score 0.055).	0.802		
TJP1-PRKCE						yes (score 0.800).	yes (score 0.808).	0.960		
TJP1-YES							yes (score 0.390). In addition, putative homologs are mentioned together in other species (score 0.218).	0.502		
TJP1-SRC					yes (score 0.545).		yes (score 0.137). In addition, putative homologs are mentioned together in other species (score 0.065).	0.600	Detected by Affinity Capture-MS assay	16944923
TJP1-ILK					none, but putative homologs were found interacting in other species (score 0.073).		yes (score 0.416). In addition, putative homologs are mentioned together in other species (score 0.063).	0.448		
TJP1-GRB2							yes (score 0.407). In addition, putative homologs are mentioned together in other species (score 0.064).	0.421		

Supplementary Table 4: List of genes included in the EMT signature "HALLMARK_EPITHELIAL_MESENCHYMAL_TRANSITION" downloaded from http://software.broadinstitute.org/gsea/msigdb. See Supplementary_Table_4
