# Supplementary material for: Proteomic screening identifies the zonula occludens protein ZO-1 as a new partner for ADAM12 in invadopodia-like structures
Source: Oncotarget. 2018 Apr 20;9(30):21366–82. doi: 10.18632/oncotarget.25106 (PMC5940405; doi:10.18632/oncotarget.25106)
Supplement: Supplementary file 2 [file oncotarget-09-21366-s002.docx]

| **Supplementary Table 1 ; List of protein specifically identified in antiADAM12 immunoprecipitates.** | |  |
| --- | --- | --- |
|  |  |  |
|  | Uniprot ID | GENE NAME |
| Metalloprotease-disintegrin 12 transmembrane isoform La OS=Homo sapiens GN=ADAM12 PE=2 SV=1 | U5NE98 | ADAM12 |
| EIF4G1 protein OS=Homo sapiens GN=EIF4G1 PE=2 SV=1 | B2RU06 | EIF4G1 |
| Isoform USP25m of Ubiquitin carboxyl-terminal hydrolase 25 OS=Homo sapiens GN=USP25 | sp\|Q9UHP3-3\|UBP25 | USP25 |
| Tight junction protein 2 (Zona occludens 2), isoform CRA_a OS=Homo sapiens GN=TJP2 PE=4 SV=1 | A0A024R233 | TJP2 |
| Polyubiquitin-C (Fragment) OS=Homo sapiens GN=UBC PE=4 SV=1 | F5H2Z3 | UBC |
| Isoform B of AP-2 complex subunit alpha-1 OS=Homo sapiens GN=AP2A1 | sp\|O95782-2\|AP2A1 | AP2A1 |
| Epididymis tissue sperm binding protein Li 14m OS=Homo sapiens PE=2 SV=1 | E9KL44 | HADHA |
| HCG1994130, isoform CRA_a OS=Homo sapiens GN=hCG_1994130 PE=2 SV=1 | B2R4W8 | hCG_1994130 |
| Pleckstrin homology domain containing, family A member 5, isoform CRA_a OS=Homo sapiens GN=PLEKHA5 PE=4 SV=1 | A0A024RAY7 | PLEKHA5 |
| Tight junction protein ZO-1 OS=Homo sapiens GN=TJP1 PE=4 SV=1 | A0A087X0K9 | TJP1 |
| cDNA FLJ53366, highly similar to Probable ATP-dependent RNA helicase DDX5 (EC 3.6.1.-) OS=Homo sapiens PE=2 SV=1 | B4DN41 | DDX5 |
| Arginine--tRNA ligase, cytoplasmic OS=Homo sapiens GN=RARS PE=1 SV=2 | sp\|P54136\|SYRC | RARS |
| ATPase family AAA domain-containing protein 3A (Fragment) OS=Homo sapiens GN=ATAD3A PE=1 SV=1 | H0Y2W2 | ATAD3A |
| Dolichyl-diphosphooligosaccharide--protein glycosyltransferase subunit 1 (Fragment) OS=Homo sapiens PE=2 SV=1 | Q53EP4 | DDOST |
| Isoform 4 of Citron Rho-interacting kinase OS=Homo sapiens GN=CIT | sp\|O14578-4\|CTRO | CIT |
| MUC19 variant 12 OS=Homo sapiens GN=MUC19 PE=2 SV=1 | G3CIG0 | MUC19 |
| 40S ribosomal protein S14 OS=Homo sapiens GN=RPS14 PE=1 SV=3 | RS14_HUMAN | RPS14 |
| Isoform 2 of Serine/threonine-protein phosphatase PGAM5, mitochondrial OS=Homo sapiens GN=PGAM5 | sp\|Q96HS1-2\|PGAM5 | PGAM5 |
| Polytrophin OS=Homo sapiens GN=TROPH PE=2 SV=1 | D4YW74 | SYNE2 |
| AFG3-like protein 2 OS=Homo sapiens GN=AFG3L2 PE=1 SV=2 | AFG32 | AFG3L2 |
| cDNA FLJ54622, highly similar to Prothrombin (EC 3.4.21.5) OS=Homo sapiens PE=2 SV=1 | B4DDT3 | F2 |
| Unconventional myosin-Ic OS=Homo sapiens GN=MYO1C PE=1 SV=1 | F5H6E2 | MYO1C |
| Isoform Short of Delta-1-pyrroline-5-carboxylate synthase OS=Homo sapiens GN=ALDH18A1 | sp\|P54886-2\|P5CS | ALDH18A1 |
| Isoform 2 of TBC1 domain family member 15 OS=Homo sapiens GN=TBC1D15 | sp\|Q8TC07-2\|TBC15 | TBC1D15 |
| Isoform Numa-m of Nuclear mitotic apparatus protein 1 OS=Homo sapiens GN=NUMA1 | sp\|Q14980-3\|NUMA1 | NUMA1 |
| Probable ATP-dependent RNA helicase DDX17 OS=Homo sapiens GN=DDX17 PE=1 SV=1 | H3BLZ8 | DDX17 |
| Melanoma inhibitory activity protein 3 OS=Homo sapiens GN=MIA3 PE=1 SV=1 | sp\|Q5JRA6\|MIA3 | MIA3 |
| Complement C4-B OS=Homo sapiens GN=C4B PE=1 SV=2 | CO4B | C4B |
| Ribosomal protein S9, isoform CRA_a OS=Homo sapiens GN=RPS9 PE=3 SV=1 | A0A024R4M0_HUMAN (+4) | RPS9 |
| cDNA FLJ57604, highly similar to GMP synthase (glutamine-hydrolyzing) (EC 6.3.5.2) OS=Homo sapiens PE=2 SV=1 | B4DUT7 | GMPS |
| Isoform 2 of Leucine-rich repeat flightless-interacting protein 1 OS=Homo sapiens GN=LRRFIP1 | sp\|Q32MZ4-2\|LRRF1 | LRRFIP1 |
| cDNA, FLJ96812, highly similar to Homo sapiens threonyl-tRNA synthetase (TARS), mRNA OS=Homo sapiens PE=2 SV=1 | B2RDX5 | TARS |
| ATP synthase subunit O, mitochondrial OS=Homo sapiens GN=ATP5O PE=1 SV=1 | ATPO | ATP5O |
| CYTSA protein OS=Homo sapiens GN=CYTSA PE=2 SV=1 | B2RMV2 | SPECC1L |
| cDNA FLJ44920 fis, clone BRAMY3011501, highly similar to Heterogeneous nuclear ribonucleoprotein U OS=Homo sapiens PE=2 SV=1 | B3KX72 | hnRNPU |
| Complement C5 OS=Homo sapiens GN=C5 PE=1 SV=4 | CO5 | C5 |
| HCG20716 OS=Homo sapiens GN=RPS26 PE=4 SV=1 | A0A024RB14 | RPS26 |
| Adenosylhomocysteinase OS=Homo sapiens GN=AHCY PE=1 SV=4 | sp\|P23526\|SAHH | AHCY |
| Isoform 2 of Nucleolar RNA helicase 2 OS=Homo sapiens GN=DDX21 | sp\|Q9NR30-2\|DDX21 | DDX21 |
| RNA-binding protein EWS OS=Homo sapiens GN=EWSR1 PE=1 SV=1 | B0QYK0 | EWSR1 |
| Isoform 4 of Zinc finger CCCH domain-containing protein 14 OS=Homo sapiens GN=ZC3H14 | sp\|Q6PJT7-4\|ZC3HE | ZC3H14 |
| MYO1B variant protein OS=Homo sapiens GN=MYO1B PE=2 SV=1 | B0I1S9 | MYO1B |
| Hypoxia up-regulated protein 1 OS=Homo sapiens GN=HYOU1 PE=4 SV=1 | A0A087X054 | HYOU1 |
| 60S ribosomal protein L14 OS=Homo sapiens GN=RPL14 PE=1 SV=4 | RL14_HUMAN | RPL14 |
| Isoform 3 of Peroxiredoxin-5, mitochondrial OS=Homo sapiens GN=PRDX5 | sp\|P30044-3\|PRDX5 | PRDX5 |
| cDNA FLJ56108, highly similar to Puromycin-sensitive aminopeptidase (EC 3.4.11.-) OS=Homo sapiens PE=2 SV=1 | B7Z4B2 | NPEPPS |
| 60S ribosomal protein L6 OS=Homo sapiens GN=RPL6 PE=2 SV=1 | Q8N5Z7_HUMAN | RPL6 |
| sp\|Q96BY6\|DOC10_HUMAN-DECOY Dedicator of cytokinesis protein 10 OS=Homo sapiens GN=DOCK10... | sp\|Q96BY6\|DOC10-DECOY | DOCK10 |
| Nuclear pore complex protein Nup205 OS=Homo sapiens GN=NUP205 PE=1 SV=3 | NU205 | NUP205 |
| ATP-dependent RNA helicase DDX3X OS=Homo sapiens GN=DDX3X PE=2 SV=1 | B5BTY4 | DDX3X |
| 40S ribosomal protein S27 OS=Homo sapiens GN=LOC392748 PE=3 SV=1 | A4D1G5_HUMAN (+3) | LOC392748 |
| cDNA FLJ50996, highly similar to 60S ribosomal protein L4 OS=Homo sapiens PE=2 SV=1 | B4DMJ6_HUMAN (+3) | NUP205 |
| DNA replication licensing factor MCM6 OS=Homo sapiens GN=MCM6 PE=1 SV=1 | MCM6 | MCM6 |
| Vacuolar protein sorting 35 variant (Fragment) OS=Homo sapiens PE=2 SV=1 | Q53FR4 | VPS35 |
| Aminopeptidase B OS=Homo sapiens GN=RNPEP PE=1 SV=1 | A6NKB8 | RNPEP |
| cDNA, FLJ95242, highly similar to Homo sapiens L-3-hydroxyacyl-Coenzyme A dehydrogenase, short chain (HADHSC), mRNA OS=Homo sapiens PE=2 SV=1 | B2RB06 | HADH |
| 60S ribosomal protein L23a OS=Homo sapiens GN=RPL23A PE=1 SV=1 | RL23A_HUMAN | RPL23A |
| cDNA FLJ46199 fis, clone TESTI4007965, highly similar to AP-1 complex subunit gamma-1 OS=Homo sapiens PE=2 SV=1 | B3KXW5 | AP1G1 |
| Retinal cone rhodopsin-sensitive cGMP 3',5'-cyclic phosphodiesterase subunit gamma OS=Homo sapiens GN=PDE6H PE=2 SV=1 | B7Z6Z4 | PDE6H |
| Inositol 1,4,5-trisphosphate receptor type 3 OS=Homo sapiens GN=ITPR3 PE=1 SV=2 | ITPR3 | ITPR3 |
| Lon protease homolog, mitochondrial OS=Homo sapiens GN=LONP1 PE=2 SV=1 | B3KU28 | LONP1 |
| Nuclear factor of activated T-cells 5, tonicity-responsive, isoform CRA_b OS=Homo sapiens GN=NFAT5 PE=4 SV=1 | A0A024R734 | NFAT5 |
| cDNA FLJ51435, moderately similar to Cofilin-1 OS=Homo sapiens PE=2 SV=1 | B4E112 | CFL1 |
| Isoform 2 of ATP-dependent 6-phosphofructokinase, platelet type OS=Homo sapiens GN=PFKP | sp\|Q01813-2\|PFKAP | PFKP |
| Coatomer subunit beta OS=Homo sapiens GN=COPB1 PE=1 SV=3 | COPB | COPB1 |
| 40S ribosomal protein S8 OS=Homo sapiens GN=RPS8 PE=2 SV=1 | Q5JR94_HUMAN (+3) | RPS8 |
| 40S ribosomal protein S2 OS=Homo sapiens GN=RPS2 PE=1 SV=1 | E9PQD7_HUMAN (+5) | RPS2 |
