# Supplementary material for: Proteomic screening identifies the zonula occludens protein ZO-1 as a new partner for ADAM12 in invadopodia-like structures
Source: Oncotarget. 2018 Apr 20;9(30):21366–82. doi: 10.18632/oncotarget.25106 (PMC5940405; doi:10.18632/oncotarget.25106)
Supplement: Supplementary file 3 [file oncotarget-09-21366-s003.docx]

| **Supplementary Table S4: List of genes included in the EMT signature "HALLMARK_EPITHELIAL_MESENCHYMAL_TRANSITION"** | | | | | | | | | |
| --- | --- | --- | --- | --- | --- | --- | --- | --- | --- |
| **downloaded from http://software.broadinstitute.org/gsea/msigdb** | | | | | |  |  |  |  |
| gene Name |  |  |  |  |  |  |  |  |  |
| ABI3BP |  |  |  |  |  |  |  |  |  |
| ACTA2 |  |  |  |  |  |  |  |  |  |
| ADAM12 |  |  |  |  |  |  |  |  |  |
| ANPEP |  |  |  |  |  |  |  |  |  |
| APLP1 |  |  |  |  |  |  |  |  |  |
| AREG |  |  |  |  |  |  |  |  |  |
| BASP1 |  |  |  |  |  |  |  |  |  |
| BDNF |  |  |  |  |  |  |  |  |  |
| BGN |  |  |  |  |  |  |  |  |  |
| BMP1 |  |  |  |  |  |  |  |  |  |
| CADM1 |  |  |  |  |  |  |  |  |  |
| CALD1 |  |  |  |  |  |  |  |  |  |
| CALU |  |  |  |  |  |  |  |  |  |
| CAP2 |  |  |  |  |  |  |  |  |  |
| CAPG |  |  |  |  |  |  |  |  |  |
| CD44 |  |  |  |  |  |  |  |  |  |
| CD59 |  |  |  |  |  |  |  |  |  |
| CDH11 |  |  |  |  |  |  |  |  |  |
| CDH2 |  |  |  |  |  |  |  |  |  |
| CDH6 |  |  |  |  |  |  |  |  |  |
| COL11A1 |  |  |  |  |  |  |  |  |  |
| COL12A1 |  |  |  |  |  |  |  |  |  |
| COL16A1 |  |  |  |  |  |  |  |  |  |
| COL1A1 |  |  |  |  |  |  |  |  |  |
| COL1A2 |  |  |  |  |  |  |  |  |  |
| COL3A1 |  |  |  |  |  |  |  |  |  |
| COL4A1 |  |  |  |  |  |  |  |  |  |
| COL4A2 |  |  |  |  |  |  |  |  |  |
| COL5A1 |  |  |  |  |  |  |  |  |  |
| COL5A2 |  |  |  |  |  |  |  |  |  |
| COL5A3 |  |  |  |  |  |  |  |  |  |
| COL6A2 |  |  |  |  |  |  |  |  |  |
| COL6A3 |  |  |  |  |  |  |  |  |  |
| COL7A1 |  |  |  |  |  |  |  |  |  |
| COL8A2 |  |  |  |  |  |  |  |  |  |
| COMP |  |  |  |  |  |  |  |  |  |
| COPA |  |  |  |  |  |  |  |  |  |
| CRLF1 |  |  |  |  |  |  |  |  |  |
| CTGF |  |  |  |  |  |  |  |  |  |
| CTHRC1 |  |  |  |  |  |  |  |  |  |
| CXCL1 |  |  |  |  |  |  |  |  |  |
| CXCL12 |  |  |  |  |  |  |  |  |  |
| CXCL6 |  |  |  |  |  |  |  |  |  |
| CYR61 |  |  |  |  |  |  |  |  |  |
| DAB2 |  |  |  |  |  |  |  |  |  |
| DCN |  |  |  |  |  |  |  |  |  |
| DKK1 |  |  |  |  |  |  |  |  |  |
| DPYSL3 |  |  |  |  |  |  |  |  |  |
| DST |  |  |  |  |  |  |  |  |  |
| ECM1 |  |  |  |  |  |  |  |  |  |
| ECM2 |  |  |  |  |  |  |  |  |  |
| EDIL3 |  |  |  |  |  |  |  |  |  |
| EFEMP2 |  |  |  |  |  |  |  |  |  |
| ELN |  |  |  |  |  |  |  |  |  |
| EMP3 |  |  |  |  |  |  |  |  |  |
| ENO2 |  |  |  |  |  |  |  |  |  |
| FAP |  |  |  |  |  |  |  |  |  |
| FAS |  |  |  |  |  |  |  |  |  |
| FBLN1 |  |  |  |  |  |  |  |  |  |
| FBLN2 |  |  |  |  |  |  |  |  |  |
| FBLN5 |  |  |  |  |  |  |  |  |  |
| FBN1 |  |  |  |  |  |  |  |  |  |
| FBN2 |  |  |  |  |  |  |  |  |  |
| FERMT2 |  |  |  |  |  |  |  |  |  |
| FGF2 |  |  |  |  |  |  |  |  |  |
| FLNA |  |  |  |  |  |  |  |  |  |
| FMOD |  |  |  |  |  |  |  |  |  |
| FN1 |  |  |  |  |  |  |  |  |  |
| FOXC2 |  |  |  |  |  |  |  |  |  |
| FSTL1 |  |  |  |  |  |  |  |  |  |
| FSTL3 |  |  |  |  |  |  |  |  |  |
| FUCA1 |  |  |  |  |  |  |  |  |  |
| FZD8 |  |  |  |  |  |  |  |  |  |
| GADD45A |  |  |  |  |  |  |  |  |  |
| GADD45B |  |  |  |  |  |  |  |  |  |
| GAS1 |  |  |  |  |  |  |  |  |  |
| GEM |  |  |  |  |  |  |  |  |  |
| GJA1 |  |  |  |  |  |  |  |  |  |
| GLIPR1 |  |  |  |  |  |  |  |  |  |
| GLT25D1 |  |  |  |  |  |  |  |  |  |
| GPC1 |  |  |  |  |  |  |  |  |  |
| GPX7 |  |  |  |  |  |  |  |  |  |
| GREM1 |  |  |  |  |  |  |  |  |  |
| HTRA1 |  |  |  |  |  |  |  |  |  |
| ID2 |  |  |  |  |  |  |  |  |  |
| IGFBP2 |  |  |  |  |  |  |  |  |  |
| IGFBP3 |  |  |  |  |  |  |  |  |  |
| IGFBP4 |  |  |  |  |  |  |  |  |  |
| IL15 |  |  |  |  |  |  |  |  |  |
| IL32 |  |  |  |  |  |  |  |  |  |
| IL6 |  |  |  |  |  |  |  |  |  |
| IL8 |  |  |  |  |  |  |  |  |  |
| INHBA |  |  |  |  |  |  |  |  |  |
| ITGA2 |  |  |  |  |  |  |  |  |  |
| ITGA5 |  |  |  |  |  |  |  |  |  |
| ITGAV |  |  |  |  |  |  |  |  |  |
| ITGB1 |  |  |  |  |  |  |  |  |  |
| ITGB3 |  |  |  |  |  |  |  |  |  |
| ITGB5 |  |  |  |  |  |  |  |  |  |
| JUN |  |  |  |  |  |  |  |  |  |
| LAMA1 |  |  |  |  |  |  |  |  |  |
| LAMA2 |  |  |  |  |  |  |  |  |  |
| LAMA3 |  |  |  |  |  |  |  |  |  |
| LAMC1 |  |  |  |  |  |  |  |  |  |
| LAMC2 |  |  |  |  |  |  |  |  |  |
| LEPRE1 |  |  |  |  |  |  |  |  |  |
| LGALS1 |  |  |  |  |  |  |  |  |  |
| LOX |  |  |  |  |  |  |  |  |  |
| LOXL1 |  |  |  |  |  |  |  |  |  |
| LOXL2 |  |  |  |  |  |  |  |  |  |
| LRP1 |  |  |  |  |  |  |  |  |  |
| LRRC15 |  |  |  |  |  |  |  |  |  |
| LUM |  |  |  |  |  |  |  |  |  |
| MAGEE1 |  |  |  |  |  |  |  |  |  |
| MATN2 |  |  |  |  |  |  |  |  |  |
| MATN3 |  |  |  |  |  |  |  |  |  |
| MCM7 |  |  |  |  |  |  |  |  |  |
| MEST |  |  |  |  |  |  |  |  |  |
| MFAP5 |  |  |  |  |  |  |  |  |  |
| MGP |  |  |  |  |  |  |  |  |  |
| MMP1 |  |  |  |  |  |  |  |  |  |
| MMP14 |  |  |  |  |  |  |  |  |  |
| MMP2 |  |  |  |  |  |  |  |  |  |
| MMP3 |  |  |  |  |  |  |  |  |  |
| MSX1 |  |  |  |  |  |  |  |  |  |
| MXRA5 |  |  |  |  |  |  |  |  |  |
| MYL9 |  |  |  |  |  |  |  |  |  |
| MYLK |  |  |  |  |  |  |  |  |  |
| NID2 |  |  |  |  |  |  |  |  |  |
| NNMT |  |  |  |  |  |  |  |  |  |
| NOTCH2 |  |  |  |  |  |  |  |  |  |
| NT5E |  |  |  |  |  |  |  |  |  |
| NTM |  |  |  |  |  |  |  |  |  |
| OXTR |  |  |  |  |  |  |  |  |  |
| PCOLCE |  |  |  |  |  |  |  |  |  |
| PCOLCE2 |  |  |  |  |  |  |  |  |  |
| PDGFRB |  |  |  |  |  |  |  |  |  |
| PDLIM4 |  |  |  |  |  |  |  |  |  |
| PFN2 |  |  |  |  |  |  |  |  |  |
| PLAUR |  |  |  |  |  |  |  |  |  |
| PLOD1 |  |  |  |  |  |  |  |  |  |
| PLOD2 |  |  |  |  |  |  |  |  |  |
| PLOD3 |  |  |  |  |  |  |  |  |  |
| PMEPA1 |  |  |  |  |  |  |  |  |  |
| PMP22 |  |  |  |  |  |  |  |  |  |
| POSTN |  |  |  |  |  |  |  |  |  |
| PPIB |  |  |  |  |  |  |  |  |  |
| PRRX1 |  |  |  |  |  |  |  |  |  |
| PRSS2 |  |  |  |  |  |  |  |  |  |
| PTHLH |  |  |  |  |  |  |  |  |  |
| PTX3 |  |  |  |  |  |  |  |  |  |
| PVR |  |  |  |  |  |  |  |  |  |
| QSOX1 |  |  |  |  |  |  |  |  |  |
| RGS4 |  |  |  |  |  |  |  |  |  |
| RHOB |  |  |  |  |  |  |  |  |  |
| SAT1 |  |  |  |  |  |  |  |  |  |
| SCG2 |  |  |  |  |  |  |  |  |  |
| SDC1 |  |  |  |  |  |  |  |  |  |
| SDC4 |  |  |  |  |  |  |  |  |  |
| SERPINE1 |  |  |  |  |  |  |  |  |  |
| SERPINE2 |  |  |  |  |  |  |  |  |  |
| SERPINH1 |  |  |  |  |  |  |  |  |  |
| SFRP1 |  |  |  |  |  |  |  |  |  |
| SFRP4 |  |  |  |  |  |  |  |  |  |
| SGCB |  |  |  |  |  |  |  |  |  |
| SGCD |  |  |  |  |  |  |  |  |  |
| SGCG |  |  |  |  |  |  |  |  |  |
| SLC6A8 |  |  |  |  |  |  |  |  |  |
| SLIT2 |  |  |  |  |  |  |  |  |  |
| SLIT3 |  |  |  |  |  |  |  |  |  |
| SNAI2 |  |  |  |  |  |  |  |  |  |
| SNTB1 |  |  |  |  |  |  |  |  |  |
| SPARC |  |  |  |  |  |  |  |  |  |
| SPOCK1 |  |  |  |  |  |  |  |  |  |
| SPP1 |  |  |  |  |  |  |  |  |  |
| TAGLN |  |  |  |  |  |  |  |  |  |
| TFPI2 |  |  |  |  |  |  |  |  |  |
| TGFB1 |  |  |  |  |  |  |  |  |  |
| TGFBI |  |  |  |  |  |  |  |  |  |
| TGFBR3 |  |  |  |  |  |  |  |  |  |
| TGM2 |  |  |  |  |  |  |  |  |  |
| THBS1 |  |  |  |  |  |  |  |  |  |
| THBS2 |  |  |  |  |  |  |  |  |  |
| THY1 |  |  |  |  |  |  |  |  |  |
| TIMP1 |  |  |  |  |  |  |  |  |  |
| TIMP3 |  |  |  |  |  |  |  |  |  |
| TNC |  |  |  |  |  |  |  |  |  |
| TNFAIP3 |  |  |  |  |  |  |  |  |  |
| TNFRSF11B |  |  |  |  |  |  |  |  |  |
| TNFRSF12A |  |  |  |  |  |  |  |  |  |
| TPM1 |  |  |  |  |  |  |  |  |  |
| TPM2 |  |  |  |  |  |  |  |  |  |
| TPM4 |  |  |  |  |  |  |  |  |  |
| VCAM1 |  |  |  |  |  |  |  |  |  |
| VCAN |  |  |  |  |  |  |  |  |  |
| VEGFA |  |  |  |  |  |  |  |  |  |
| VEGFC |  |  |  |  |  |  |  |  |  |
| VIM |  |  |  |  |  |  |  |  |  |
| WIPF1 |  |  |  |  |  |  |  |  |  |
| WNT5A |  |  |  |  |  |  |  |  |  |
